# Implications of uncertainty in technology cost projections for least-cost decarbonized electricity systems

**DOI:** 10.1016/j.isci.2023.108685

**Published:** 2023-12-07

**Authors:** Lei Duan, Ken Caldeira

**Affiliations:** 1 Department of Global Ecology, Carnegie Institution for Science, Stanford, CA 94305, USA; 2 Orca Sciences LLC, Kirkland, WA 98033, USA; 3 Breakthrough Energy LLC, Kirkland, WA 98033, USA

**Keywords:** Applied sciences, Energy management, Energy Modelling

## Abstract

Plans for decarbonized electricity systems rely on projections of highly uncertain future technology costs. We use a stylized model to investigate the influence of future cost uncertainty, as represented by different projections in the National Renewable Energy Laboratory 2021 Annual Technology Baseline dataset, on technology mixes comprising least-cost decarbonized electricity systems. Our analysis shows that given the level of future cost uncertainty as represented by these projections, it is not possible to predict with confidence which technologies will play a dominant role in future least-cost carbon emission–free energy systems. Successful efforts to reduce costs of individual technologies may or may not lead to system cost reductions and widespread deployments, depending on the success of cost-reduction efforts for competing and complementary technologies. These results suggest a portfolio approach to reducing technology costs. Reliance on uncertain cost breakthroughs risks costly outcomes. Iterative decision-making with learning can help mitigate these risks.

## Highlights

• Future cost uncertainties lead to different least-cost decarbonized energy systems
• Cost reductions in one technology do not guarantee more uses of that technology
• Cheaper onshore wind and geothermal reduce uncertainties of energy system behaviors
• Relying on uncertain cost breakthroughs risks costly outcomes

## Graphical abstract

**Figure:**
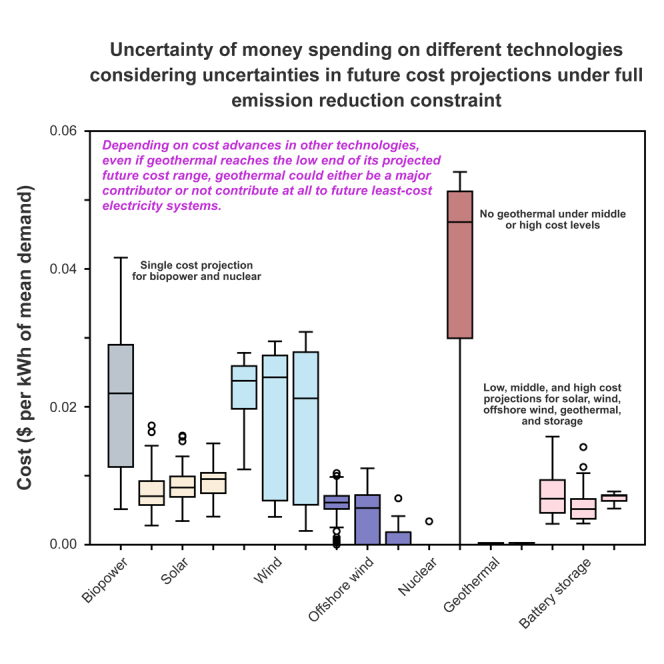

## Introduction

Stabilizing global mean temperature and reducing adverse consequences of the climate change caused by increasing concentration of atmospheric carbon dioxide (CO_2_) motivate rapid decarbonization of the entire economy.^1^^,^^2^ Decarbonizing electricity generation plays a crucial role in limiting anthropogenic carbon emissions, a role that grows more important with the electrification of other sectors, such as construction, transportation, and industry.^3^^,^^4^^,^^5^^,^^6^^,^^7^^,^^8^^,^^9^ Legislation and commitments to decarbonize have been proposed in multiple regions, and many of them specify that electricity will be generated with zero-carbon emission by the year 2050 or earlier.^10^^,^^11^^,^^12^^,^^13^^,^^14^ To economically decarbonize the electricity sector, no- or low-carbon emission generation technologies with reduced costs are needed to avoid emissions from fossil fuel sources (e.g., coal, oil, and natural gas).

Studies of decarbonized electricity systems often consider a portfolio of low-carbon emission technologies with prominently different characteristics,^4^^,^^9^^,^^15^^,^^16^^,^^17^^,^^18^^,^^19^^,^^20^^,^^21^^,^^22^^,^^23^^,^^24^ and often use an ad hoc set of assumptions for future costs as chosen by the author teams. Among these technologies, variable renewables—mainly onshore wind (denoted as wind to distinguish from the offshore wind hereafter) and solar photovoltaics (solar) generation—harness renewable energy inputs from nature and have no fuel cost. Costs of wind and solar have substantially decreased over the past decade and are projected to continue decreasing in the future,^25^ making them economically competitive in global electricity markets.^19^^,^^26^^,^^27^^,^^28^ Meanwhile, realizing deep decarbonization of the electricity systems that rely primarily on wind and solar suggest to be much more challenging than realizing comparatively modest emission reduction scenarios due to their variability.^18^^,^^29^^,^^30^^,^^31^^,^^32^^,^^33^ In contrast, the most notable low-carbon emission firm technology, the nuclear power plant, is less strongly affected by weather conditions, and can generate stable power outputs. However, many other concerns (safety issue, waste disposal, etc.) have resulted in increased costs and moderated policy support for nuclear power, and led to early retirements of nuclear power plants in some regions.^34^^,^^35^

Modeling studies that explore pathways to cost effectively decarbonize electricity systems rely on cost estimates or learning-rate assumptions for technologies as inputs to models. Cost estimates for different electricity generation and storage technologies covering periods from the near-current to the year 2050, for the United States (US) for example, have been developed by various research groups.^36^^,^^37^^,^^38^ Whereas near-current technology cost estimates are similar to one another, future cost projections diverge and are much more uncertain. Projected technology costs made by different groups or different trajectories within the same group vary due to differences in assumptions, such as levels of confidence in future technology innovations. Projected technology costs made by the same group also vary with time as a result of updated underlying assumptions with improved understanding. Different future cost projections, along with uncertainties in other components, could have substantial impact on the simulations of relative competitiveness among low-carbon emission technologies, especially under a cost-minimizing framework.^39^^,^^40^^,^^41^

Modeling studies considering different cost inputs might lead to different conclusions about the composition of cost-effective decarbonized electricity systems.^4^^,^^18^^,^^20^^,^^21^^,^^29^^,^^42^^,^^43^^,^^44^^,^^45^^,^^46^ For example, studies that assume substantial cost reductions in variable renewables with no or little improvements in nuclear power have concluded that least-cost zero-emission electricity systems may rely primarily on wind and solar, often based on the assumption that low-cost energy storage and load response will be available to provide reliable delivery of energy services.^45^^,^^46^^,^^47^^,^^48^^,^^49^ Other studies considering cost reduction possibilities of more technologies often show additional benefits by including low-carbon emission firm generation (e.g., nuclear, biopower, and geothermal).^18^^,^^20^^,^^21^

Previous studies have explored the potential impact of technology cost uncertainties in modeling decarbonized energy systems.^40^^,^^50^^,^^51^^,^^52^^,^^53^^,^^54^ For example, Neumann and Brown (2023) considered cost uncertainties on renewable technologies and identified ranges of cost-efficient capacity expansion plans for European electricity systems; Pilpola and Lund (2019)^40^ applied a Monte Carlo approach on Finnish energy systems and emphasized the importance of addressing input uncertainties in future low-carbon-emission energy system planning. Most of these studies, however, embedded the cost uncertainty within a wide range of sources of uncertainty, such as the level of electricity consumption and renewable resource availability, and they focused primarily on system-level behaviors including the annual system cost, CO_2_ emissions, and total power supplied. It remains unclear about the sole impact of future cost uncertainty on system dynamics and the resulted technology-mix in least-cost carbon-emission-free energy systems. Given the urgent need to eliminate carbon emissions and the fact that power sector capital equipment, once built, will remain in place for a long time and have long-lasting effect, it is important to understand the impact of uncertainty in future technology cost projections on the composition of least-cost decarbonized electricity systems.

In this study, we investigate how different technology cost projections could affect simulations of the technology mix of future decarbonized electricity systems. As we will show, given the level of future cost uncertainty, it is not possible to predict with confidence which technologies will play a dominant role in a future least-cost carbon emission–free energy system, and cost reduction in certain technologies do not necessarily guarantee the increased deployments of those technologies.

**Figure 1:**
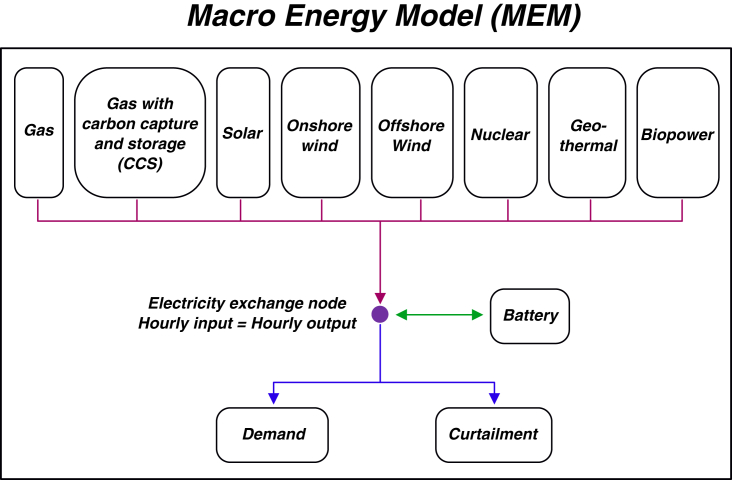
Diagram of the linear-optimization electricity system model used in this analysis Wind and solar fields represent variability in the contiguous US. Cost assumptions are from the National Renewable Energy Laboratory (NREL) 2021 Annual Technology Baseline (ATB) dataset.

Here, we use the macro energy model (MEM).^23^^,^^24^^,^^47^^,^^49^^,^^55^ MEM is constructed as a linear-optimization model that considers only techno-economic factors. The objective function for minimization is the total system cost associated with capacity expansion and power dispatch of a portfolio of generation and storage technologies (see Figure 1 for technologies considered in the main cases). A portfolio approach is thought to provide economic and technical benefits^56^^,^^57^ and here to better capture the impact of cost variation of a wider range of technologies. Electricity provided to the exchange node is used to meet electricity demand, or curtailed as appropriate. Here, we conduct a series of single-year optimizations with hourly time resolution in a “greenfield” setting (i.e., no assumption of pre-existing capacity) and require that 100% electricity demand be satisfied at each hour. To facilitate understanding, we normalize the demand profile by dividing annual mean demand on each hourly step (i.e., annual mean demand is 1 kWh after normalization). Hourly demand and wind and solar generation potentials are calculated for the US (see STAR Methods). A fixed cost is associated with all technologies to represent the fixed capital investment (Table S1). This includes the purchase and installation costs and fixed operation and maintenance (O&M) costs. A variable cost is specified for gas, gas with carbon capture and storage (gas-with-CCS), nuclear, and biopower. This variable cost includes variable O&M costs and fuel costs as appropriate. For nuclear, we assume that the nuclear reactor must be operated at constant rates, and thus operation and fuel costs (i.e., variable costs) are added to the fixed cost as dollar per unit capacity.

A common problem in assessing effects of cost uncertainty on future least-cost system is attaining some consensus on the probability distribution of future technology costs or learning rates. Here, we aim to illustrate the role of cost uncertainty without making claims regarding future probabilities ourselves. Therefore, we adopt near-current (i.e., year 2019) and future (e.g., year 2050) cost estimates for technologies in the main cases based on the National Renewable Energy Laboratory (NREL) 2021 Annual Technology Baseline (ATB) dataset.^37^ We consider single cost estimates for all technologies for the year 2019 cases, and three cost estimates for future-year cases for each technology except for gas, biopower, and nuclear, which have one cost projection in the NREL 2021 ATB dataset. With these reduction pathways, we aim to represent the advanced, moderate, and conservative technology innovation pathways, which lead to low, middle, and high future cost levels, respectively. Numbers from the 2021 ATB dataset are converted to cost inputs for MEM in a consistent manner for all technologies. That is, we do not generate any cost estimates ourselves, but rely entirely on cost projections from the NREL ATB dataset. To isolate the impact of different cost projections, we compare simulations using the same electricity demand and resource profiles. We analyze six technologies with three cost projections (i.e., gas-with-CCS, solar, wind, offshore wind, geothermal, and battery storage). This leads to a total of 729 possible cost combinations (243 combinations under 100% emission reduction scenarios since we focuse on eliminating carbon emissions from gas and gas-with-CCS). The wide range of cost combinations facilitates our understandings of the system dynamics under different technology innovation scenarios. Where a probability assessment is required for illustrative purposes, we consider these cost projections to be equally probable. Caveats of these assumptions are discussed in limitations of the study.

Our analysis provides an idealized and transparent framework. We do not claim to have predictive skill in projecting technology costs. To illustrate the influence of cost uncertainty, we rely on the NREL ATB cost projections, and focus on the fundamental understanding of systematic dynamics. Many factors could affect future electricity costs and system planning, such as the availability of innovative low-carbon emission technologies, changes in global and domestic policy conditions, market responses, etc., which are beyond the scope of this study. Numerical values presented here might change easily, while qualitative conclusions about the influence of cost uncertainty on future least-cost systems are likely robust. We provide transparent analysis with full details disclosed, and hope to inspire discussions on such topics and studies that apply more sophisticated assumptions and advanced approaches.

In the Results section, we focus on results from our main cases that consider technologies listed in Figure 1 and technology cost estimates from the NREL 2021 ATB report. In the limitations of the study section, we further cover issues such as lower future cost estimates made for renewable technologies in the past,^25^^,^^58^^,^^59^ impact of different discount rates, impact of including long-duration storage and direct air capture, using more recent future technology cost projections, other demand profiles, and other caveats associated with our stylized modeling framework. Our main findings are summarized in the discussion section.

## Results

### Electricity system under representative cost projections

We first discuss results under the NREL ATB year-2019 cost levels for the US, where we gradually enforce carbon emission reduction constraints that remove fossil fuel sources (i.e., gas and gas-with-CCS). More information can be found in Text S1. In our simulations, gas and gas-with-CCS are the primary sources of electricity dispatch when there is small cost on carbon emissions (i.e., low emission reduction constraints) (Figures S1 and S2), because they are the lowest-cost way to meet electricity demand given the 2019 cost levels. As emission reduction constraints tighten, more electricity comes from wind and solar, with flexible fossil fuel generations filling gaps between variable electricity supply and demand. Under very deep emission reduction constraints (e.g., >90% emission reduction constraints), nuclear and biopower can be least-cost providers of reliable electricity, with nuclear providing constant and stable electricity generation, and wind and solar, supported by an increasing amount of biopower and battery storage, address variability in demand. When biopower is removed from the system, nuclear becomes more competitive and dominates the deep decarbonization scenarios (Figure S3) due to the high variability of wind and solar, and high cost of batteries; when nuclear is removed as well, curtailments from wind (both onshore and offshore) and solar increase substantially under deep emission reduction constraints along with system costs. These results are consistent with previous simulations using the same model and near-current cost estimates from the Energy Information Administration (EIA).^23^ Results using year 2016 to year 2018 demand, with wind and solar potentials are shown in Figure S4.

Projected year-2050 fixed costs of all technologies show substantially different cost reductions relative to the 2019 estimates. For example, ratios of fixed costs between 2050 and 2019 are >85% for technologies such as gas, biopower, nuclear, and geothermal under high future cost projection (i.e., conservative technology innovation) trajectories, suggesting modest cost reductions. Meanwhile, these fixed cost ratios are <40% for technologies including wind, solar, storage, and geothermal under low future cost projection (i.e., advanced technology innovation) trajectories (Figure S5 and Table S1), indicating great innovations have been achieved for these technologies.

**Figure 2:**
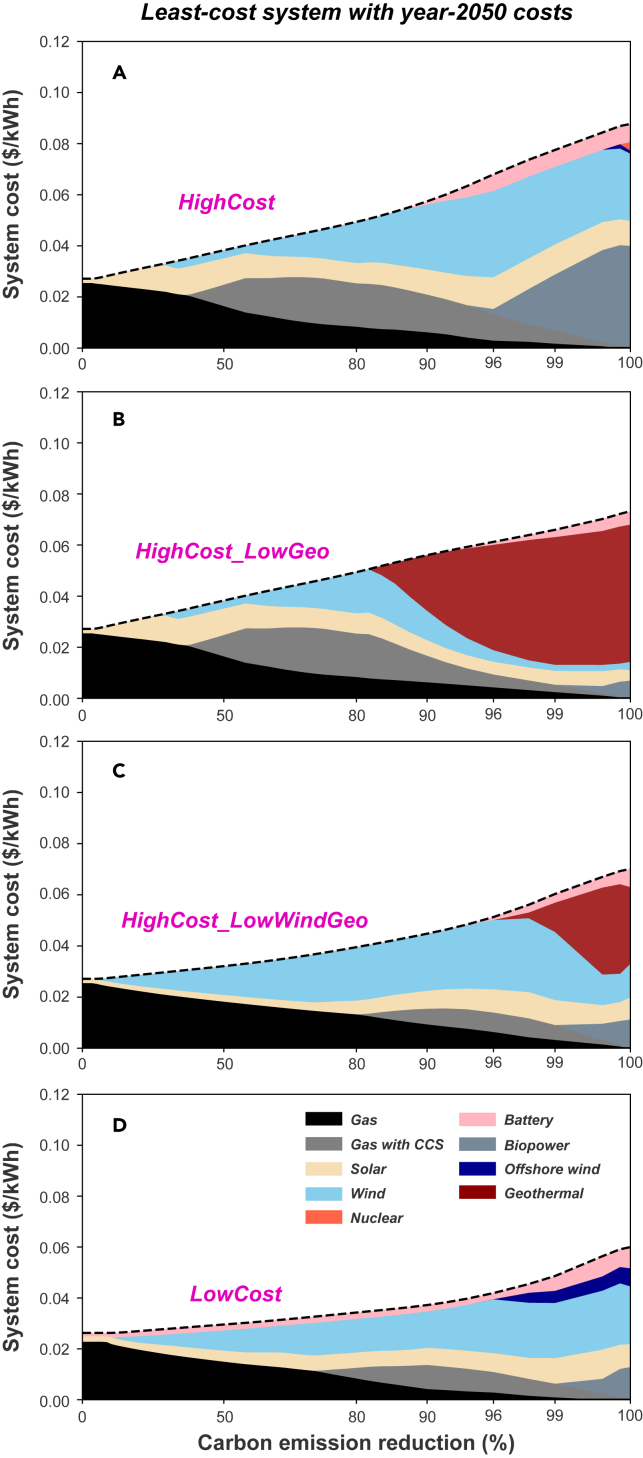
Contributions to total system costs Four illustrative cases under 2050 cost levels and various emission reduction constraints are compared. (A) **HighCost** all technologies (excluding gas, nuclear, and biopower, which have only one future cost projection) follow the high future cost projections. (B) **HighCost_LowGeo** where only geothermal reaches its low cost projection while others remain at high costs projections. (C) **HighCost_LowWindGeo** where both wind and geothermal reach their low cost projections; and (D) **LowCost** where all technologies reach their low future cost projections. The same 2019 demand and generation potential profiles are used to isolate the impact caused by applying different technology costs. Geothermal plays a major role if it is the only technology to reach its low-cost level, but if other technologies reach their low-cost levels, the role of geothermal is reduced or eliminated.

We analyze six technologies with three cost projections (i.e., gas-with-CCS, solar, wind, offshore wind, geothermal, and battery storage). This leads to a total of 729 possible cost combinations (243 combinations under 100% emission reduction scenarios because gas-with-CCS cannot participate). To facilitate analysis of different cost assumptions, four illustrative 2050 cost combination cases are selected and compared in Figure 2, in which: all these six technologies follow the high future cost projections to represent conservative innovations for all technologies (“**HighCost**”); only geothermal achieves the advanced innovations and reaches its low cost projection while other technologies remain at high costs projections (“**HighCost_LowGeo**”); both wind and geothermal reach their low cost projections (“**HighCost_LowWindGeo**”); and all technologies achieve their advanced innovations and reach their low cost projections (“**LowCost**”). **HighCost** and **LowCost** are chosen because they represent NREL ATB’s most optimistic and pessimistic technology innovation scenarios; **HighCost_LowGeo** is used to highlight the case where cost ratios between renewables and firm technologies are larger and geothermal dominates renewables under deep emission reduction constraints; and **HighCost_LowWindGeo** is used to represents the case where both renewables and geothermal contribute substantially under deep emission reduction constraints.

Several features are noticeable across these cost combination cases. First of all, all four cases share similar behaviors as they move from low to modest emission reduction constraints. For example, gas consistently acts as the least costly way to meet electricity demand when no emission reduction constraint is implemented. Under modest emission reduction constraints (e.g., <80% emission reduction constraint), low-carbon emission firm technologies cannot compete with wind and solar. Substantial cost reductions in wind and solar in 2050 lead to decreased use of gas-with-CCS compared to the 2019 results. Competition between wind and solar depends initially on their relative magnitudes of cost reductions. As emission reduction constraints are enhanced, wind becomes more important (Figures 2 and S6).

The four scenarios with representative cost combinations examined here show distinguishable outcomes under deep emission reduction constraints (e.g., >90% emission reduction constraint). For the **HighCost** case, because the 20% cost reduction for wind and >40% cost reduction for solar are larger than cost reductions for firm technologies (less than 15% for nuclear and geothermal; both of them also have substantially larger costs per kWh than wind and solar at the 2019 level), wind and solar are much more attractive and outcompete firm generations in deep emission reduction scenarios, with dispatches from biopower to fulfill flexibility purposes. In **HighCost**, nuclear is rarely used even under the 100% emission reduction constraint because costs of nuclear are projected to decline less than the costs of most competing technologies (details depend on the year of hourly demand and wind and solar profiles used for optimizations, see Figure S4).

**Figure 3:**
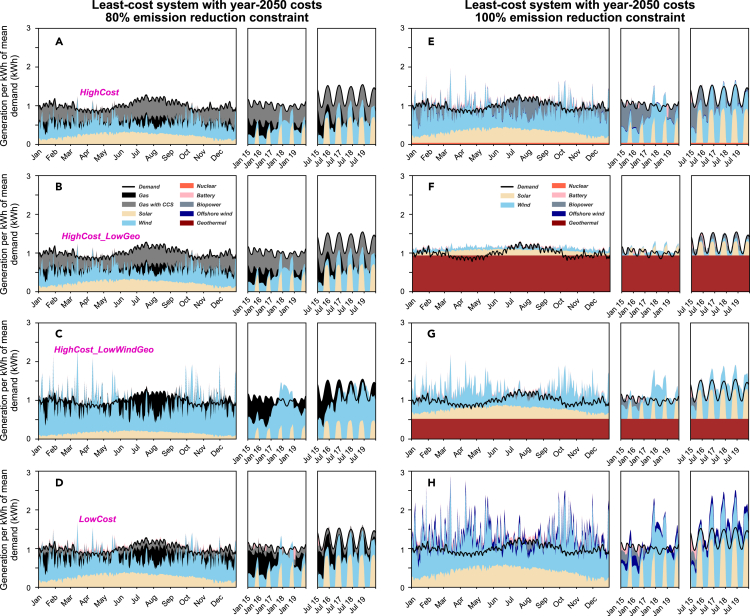
Daily and hourly electricity dispatches Four 2050 cost scenarios with different cost combinations are selected (**HighCost**, **HighCost_LowGeo**, **HighCost_LowWindGeo**, and **LowCost**) under (A–D) 80% and (E–H) 100% emission reduction constraints. Rows correspond to the four panels in Figure 2. Wind and solar consistently serve as lower-cost options for modest emission reduction constraints when fossil fuels are available to provide system reliability, whereas a combination or wind, solar and/or geothermal can dominate under deep emission reduction constraints.

When only geothermal achieves advanced cost innovations and reaches its low future cost projection (i.e., to 27% of current costs, **HighCost_LowGeo**, Figure 2B), geothermal replaces much wind and solar, and dominates systems under deep emission reduction constraints. If wind also achieves its low future cost projection (i.e., to 32% of current costs, **HighCost_LowWindGeo**, Figure 2C), the market share for geothermal is markedly reduced. If all competing technologies achieve their low future cost projections (**LowCost**, Figure 2D), then geothermal is no longer able to compete. In our analysis, when all technologies experience substantial cost reductions (**LowCost**), primarily wind, solar, and biopower, but also offshore wind and battery storage, dominate systems under deep emission reduction constraints. System costs under the same emission reduction constraints in **LowCost** are substantially lower (on average 38% lower for >90% emission reduction constraints) compared to system costs in **HighCost**. Biopower use is greatly reduced in **HighCost_LowGeo** and **HighCost_LowWindGeo** due to the reduced use of variable wind and solar generation. Biopower use is also reduced in **LowCost** because wind and solar are less costly, and this results in more curtailments (Figure 3).

### System cost distributions among ensemble members

In this section, we analyze the least-cost optimizations for all possible cost combinations at 2050 cost levels.

**Figure 4:**
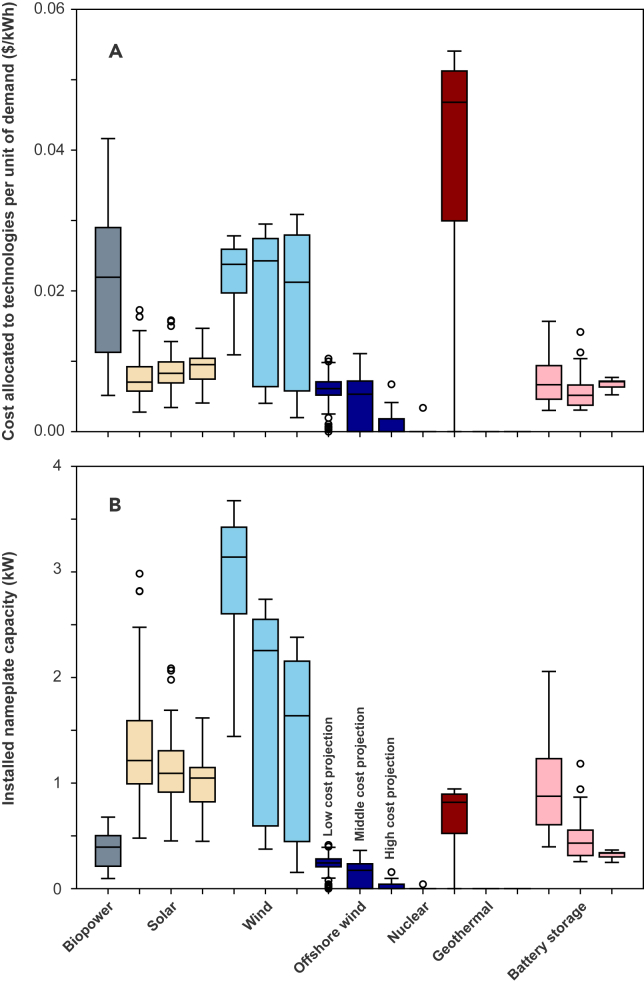
Distributions of simulated cost allocated to each technology and capacities Results of (A) system cost attributed to different technologies and (B) installed nameplate capacity (i.e., capacity associated with fixed cost; actual generation potential equals installed nameplate capacity times capacity factor for wind and solar on each time step) are plotted for each technology under various 2050 projected cost levels using the Matplotlib function Boxplot. The boxes extend from the first quartile to the third quartile of the data, with a line at the median. Whiskers extend from the box to the farthest data point lying within 1.5x the inter-quartile range from the box. In our linear-optimization framework, system cost can be represented as the linear sum of costs attributed to different technologies, including the fixed cost of installed capacity (as shown in panel B) plus non-zero variable cost of total electricity dispatched (e.g., gas and biopower). Cases under the 100% emission reduction constraint are shown, and results are expressed in terms of cost or power per kWh of annual mean demand. For technologies with three cost projections in NREL ATB, there are 81 combinations of costs of the other technologies under each cost level. Biopower and nuclear have only one cost projection. Geothermal has non-zero capacity only when it reaches its lowest cost level. Cost reductions in a technology typically result in an expectation of increases in deployed capacity of that technology, but not necessarily an expectation of an increased spending on that technology (e.g., solar).

Figure 4 shows the distribution of simulated costs and installed nameplate capacities for different technologies and cost projection levels, under the 100% emission reduction constraint (results under the 99% emission reduction constraint are plotted in Figure S7). In general, cost reductions for technologies with multiple cost projection levels lead to more penetration and installed capacity of that technology. Geothermal shows up in optimal systems only when its low future cost projection is achieved with its degree of deployment being very sensitive to the cost of wind power (Figure S8). In our simulations, cost reduction in each technology tend to increase installed capacities of that technology, but the total amount of spent on deploying that technology (in absolute terms as plotted in Figure 4 or percentages to total system costs listed in Tables S2 and S3) does not in general increase for all technologies. For example, under the 100% emission reduction constraint, contributions of solar to total system costs range from 5.6% to 19.0% under the high-cost projection, while the numbers range from 3.9% to 23.3% under the low-cost projection. Similarly, contributions of battery storage to total system costs range from 7.2% to 11.1% under the high-cost projection, while the numbers range from 4.4% to 22.3% under the low-cost projection. Figure S9 shows that solar exhibits a median price elasticity of demand (here we refer to the input fixed costs as “price” and total capacity installed as “demand” for each technology) that is less than 1.0 – for each percentage decrease in the cost of solar there is a smaller percentage increase in the solar capacity deployed. In contrast, onshore and offshore wind, and battery storage exhibit a median price elasticity of demand that is close or greater than 1.0.

**Figure 5:**
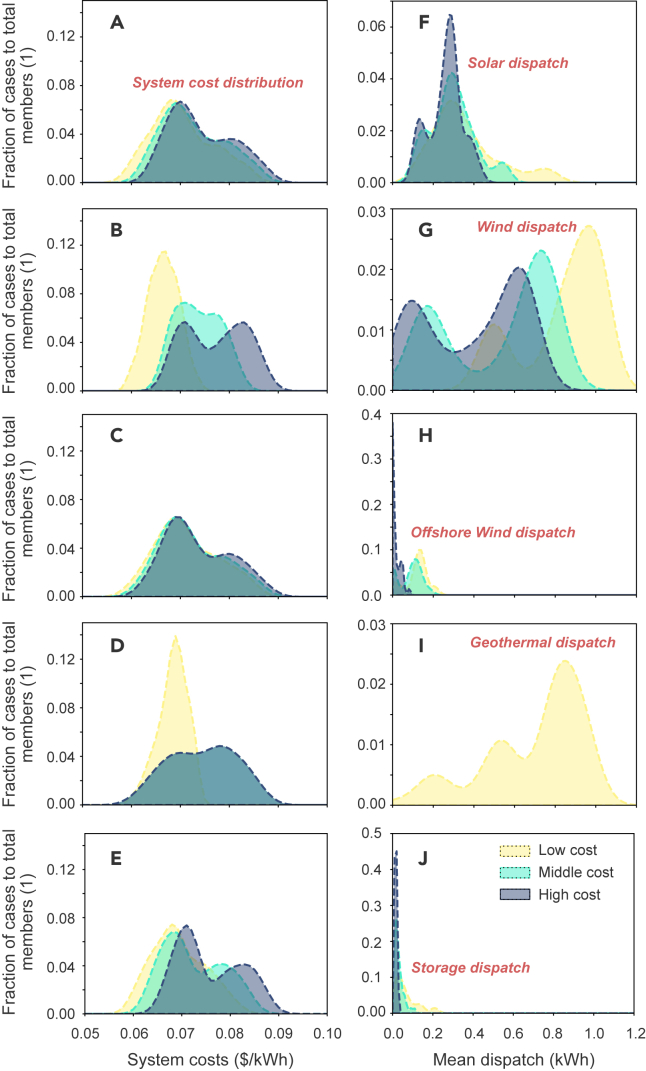
Probability distribution of ensemble members X axis represents different bins of (A–E) system costs and (F–J) mean electricity dispatch levels, and y axis shows the ratio of the number of cases in each bin to total number of ensemble members under various technology cost levels. That is, each panel includes all ensemble members and each pattern (i.e., one of the advanced, moderate, and conservative innovation trajectories) has one-third of ensemble members. Ensemble members under the 100% emission reduction constraints (in total 243 cases). The distribution function is produced using a Gaussian kernel density estimation tool from the SciPy package in Python. Cost reductions in wind and geothermal not only decrease overall system costs, but also reduce the uncertainty of system cost distributions.

Figure 5 shows the distribution of ensemble members under the 100% emission reduction constraint, that is, the ratio of cases under different system costs and mean electricity dispatch levels to total ensemble members (i.e., the number of selected cases divided by the total number of cases), as a function of technology types and cost projection levels. Results under the 99% emission reduction constraint are plotted in Figure S10. In our simulations, cost reductions in solar and offshore wind have limited impact on the distribution of system costs, with lower cost projections slightly increase their capacities in the optimized solutions. In contrast, cost reductions in wind and geothermal not only decrease overall system costs, but also reduce the uncertainty of system cost distributions (i.e., narrowing the width of the pattern). For example, system costs differ by as much as 30% when the wind cost follows its high future cost projection, and only 22% at its low future cost projection; system costs differ by 46% when the geothermal cost follows its high future cost projection, and only 22% at its low future cost projection. This occurs because the substantial cost reductions in these technologies result in a dominant role of either technology, which substantially increases their capacities and dispatches in the systems and leaves limited space for other technologies to compete. As a result, the costs of minor competing technologies have little influence on system costs.

Figure S11 further examines how important cost reductions in each technology are in affecting overall system cost distribution. Across all 243 combinations for the year 2050 under the 100% emission reduction constraint, cost reductions in wind and geothermal have the largest impact on expected system cost. In our idealized analysis, the least-cost system ($0.06/kWh) among all possible solutions (as gas and gas-with-CCS are excluded) is comprised of all technologies (except nuclear and geothermal) at their lowest cost levels (as described previously, biopower and nuclear have only one cost projection). Geothermal does not compete in the least-cost condition, but there are systems with costs no more than 5% greater than the least-cost solution, in which geothermal competes at its low future cost projection. We also observe systems with costs within 10% of the least-cost solution, that include solar, offshore wind, and battery storage, competing at their middle cost projections. Similarly, there are systems costing no more than 20% over the least-cost solution where solar competes at its high, and wind at its middle cost projections. With the exception of wind and geothermal, decrease in the cost of one technology does not greatly reduce the mean or the variation of system cost (Figure S11). That is, if we get any single one of these technologies to be low cost, we could still end up with a costly system if we fail to lower costs in other technologies. Meanwhile, system costs would be moderate knowing that wind or geothermal would be on their low-cost levels, even if we fail to lower costs of other technologies. In these cases, substantial amounts of cheap wind and/or geothermal capacities are deployed, ensuring moderate to low system costs.

In our main scenarios, we consider unlimited resource potential for all technologies, while the US Department of Energy Geothermal Technologies Office reported ∼530 GW as the total technical potential for electricity generation for all types of geothermal. This accounts for approximately 70% of the 2050 average hourly demand according to estimations from the NREL Electrification Future Study.^60^^,^^61^ To reflect such limitation on geothermal, we reanalyze the 100% emission reduction constraint cases and restrict the maximum capacity of geothermal to certain levels (i.e., 0.7, 0.5, and 0.3 times the annual mean demand). Figure S12 shows that restricting the maximum geothermal capacity, in general, results in a reduced ratio of cases under lower system costs and increases the ratio under higher costs. Among all ensemble members, restricting the capacity of geothermal has the least impact on systems with wind at its low future cost projection (Figure S13). This is because cheaper wind power directly competes with geothermal to provide the bulk of electricity, and thus there is minimal need to build geothermal with wind at its low future cost projection.

Our main cases are single-year least-cost optimizations assuming complete information, in which technology costs and emission constraints are prescribed at specific levels, and the model considers an electricity system at a stationary state without pre-existing capacity. In the real world, technology costs would evolve at different rates, and the capacity of any technology built in earlier years would last for decades and have long-term impacts. We conduct stylized transient simulations starting from 2019 untill 2050 with yearly updated emission reduction constraints and technology cost inputs. Compared to the single year 2050 optimization results, stylized transient simulations to 2050 show higher system costs and a wider distribution of system costs (Figures S14 and S15). In these cases, there are more wind, solar, offshore wind, and battery storage, and less geothermal due to the persistence into the time of 100% emission reduction constraint of capital stock that was built under weaker emission constraints (Figure S16). Detailed discussions are included in Text S2.

### Expected projected system cost and uncertainty

If someone were to plan future electricity systems based on current expectations of potential technology cost reductions and only later were able to identify which of the cost estimates are correct, which system would be preferred? This approach is intended to be analogous to situations in which energy system decisions are made based on assumptions of future cost, which may or may not be correct.

To fully explore such question is beyond the scope of our analysis. However, we can still perform high-level assessments utilizing our idealized framework. To do this, we reframe the question as: if someone were to build these systems given some NREL 2021 ATB technology cost projections, and only later were told which of the cost projections are correct, which system would have the lowest expected cost and the lowest uncertainty of expected cost? In our analysis, projected system costs under given cost reduction projections can be calculated using ensemble simulations under the 100% emission reduction constraint from the previous section and the distribution of 2050 cost combinations. That is, we take the simulated technology capacities and dispatches from each ensemble member that already exists (i.e., systems that were built based on given cost assumptions, in total 243 ensemble members) and see how the system cost would change when giving all possible cost combinations (the actual system costs, in total 243 cost combinations for each ensemble member). If we make the assumption that all of the NREL 2021 ATB cost projections are equally likely, then the expected cost for each ensemble member or certain technology capacity mix is the mean, and uncertainty can be represented as one standard deviation of system cost across all 243 cost combinations.

**Figure 6:**
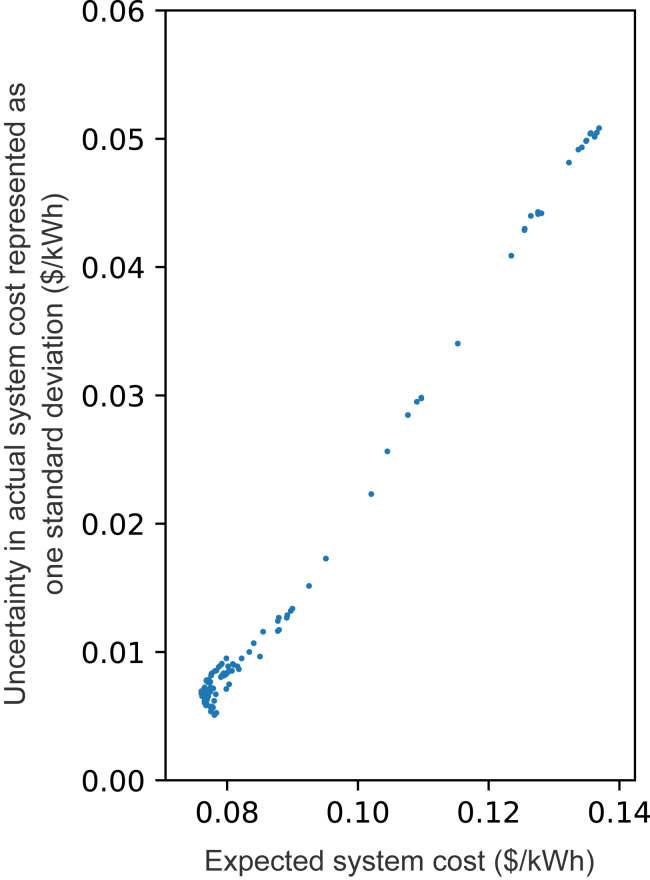
Expected system cost and uncertainty in actual cost X axis represents the expected system cost, which is calculated as the mean value of all 243 possible costs for a certain capacity mix. Y axis represents the uncertainty in actual cost, which is calculated at one standard deviation from all 243 possible costs. Here, we consider optimal technology capacity mix using ensemble members under the 100% emission reduction constraint. Possible electricity systems with lower expected costs may have lower uncertainties in realized costs.

Figure 6 shows that systems (or technology capacity mixes) that show lower expected (i.e., average) projected costs across all 243 possible cost combinations also have smaller values of one standard deviation of projected cost. For technology mixes with zero geothermal capacity, wind contributes to the largest cost difference among cost combinations (Figure S17); the presence of geothermal substantially increases both expected costs and its uncertainties due to the wider range in its projected 2050 cost reduction potentials (ranging from ∼12% to 73%), and the fact that geothermal is cost-competitive at its low future cost projection. Our results thus indicate that approaches that minimize expected costs across the probability distribution of future actual costs are more likely to produce lower cost systems than will approaches built in the hope that some current-costly technology will achieve a cost breakthrough. If the cost breakthrough fails to materialize, the resulting systems could prove costly. Here, variations in the costs of geothermal and wind have the largest influence.

## Discussion

Here, we conduct an idealized and transparent analysis to explore the impact of uncertainty in technology cost projections on the results of least-cost optimizations of future decarbonized electricity systems. We have used future cost estimates from a single source to reduce the possibility of bias that might be introduced by using an ad hoc set of assumptions made by the author team. We have shown that with the level of future cost uncertainty as represented by the NREL ATB projections, it is not possible to predict with confidence which technologies will play a dominant role in a future least-cost carbon-emission-free energy system.

Our model takes into consideration techno-economic factors only, and the objective function is designed to minimize the system cost considering technology capacity and electricity dispatch. We compare simulations that isolate the impact of applying different technology cost inputs based on future projections from the NREL 2021 ATB dataset. Our study focuses on a fundamental understanding of system dynamics. While our numerical results should not be overinterpreted, the qualitative influence of future cost uncertainty on future least-cost electricity systems will likely prevail under more generalized conditions.

Compared to the near current cost levels (i.e., 2019 estimates), fixed cost associated with construction and fixed operation and management (O&M) decreases to 2050 for all technologies examined here, with cost reductions varying from less than 15% for technologies such as gas, biopower, nuclear, and geothermal under the conservative technology innovation trajectory to more than 60% for technologies such as onshore wind, solar, battery, and geothermal under the advanced technology innovation trajectory.

Simulations considering a wide range of emission reduction constraints using different technology cost combinations indicate that optimized electricity systems are more similar under low to modest emission reduction constraints (e.g., <80% emission reduction constraint). Under these constraints, variable renewables, specifically solar and wind, are low-cost options preferred as low-carbon electricity generation, with flexible fossil fuels filling gaps at times of low variable renewable supply and/or high electricity demand. Under deep-decarbonization scenarios (e.g., >90% emission reduction constraint), however, either variable renewables (e.g., wind and solar) or firm generation technologies (i.e., geothermal) could dominate the least-cost system, depending on relative ratios of realized cost reduction.

Considering all possible cost combinations from the NREL ATB dataset under the deep emission reduction constraints (243 cases under 100% and 729 cases under 99% emission reduction constraints), the largest system cost when all technologies follow their high-cost projection levels is less than two times the least-cost solution when all technologies follow their low-cost projection levels, highlighting the benefits of using a portfolio of technologies.

Our study has shown that cost reductions of certain technologies do not guarantee an increased deployment share of those technologies, as the least-cost solution depends on cost reductions of competing and complementary technologies as well. For example, we have shown that solar and battery storage contribute to as low as 3.9% and 4.4% of total system costs under low-cost projections, smaller than that under high-cost projections (5.6% and 7.2%). We also find that different technologies exhibit different price elasticities of demand. For each percentage decrease in cost, there is a greater increase in capacity demand for technologies such as onshore wind than for technologies such as solar. Different price elasticities of demand in an energy sector might have important implications of the economic scale and growth pattern for different energy technology industries.

Among all technologies, knowing that onshore wind or geothermal would follow their low future cost projections would substantially reduce the range of simulated system costs. That is, cost reductions in both onshore wind and geothermal not only increase the probability of a less costly system, but such cost reductions also reduce the uncertainty of system cost distributions. This is because these technologies are becoming more competitive under lower cost projections than other technologies. Geothermal, with a wide range of future cost projections, shows up only in systems where its low-cost estimate is achieved, and it competes mostly with onshore wind. Meanwhile, cost reductions in technologies such as solar and offshore wind have very limited impact on the probability distribution of optimized system costs.

Placing too much trust in unproven technologies can be risky. Considering situations in which energy system decisions are made based on assumptions of future cost that may or may not be correct, our results indicate that electricity systems with lower expected costs also tend to have lower uncertainties in actual cost. Systems built in the hope that some current costly technology will achieve a cost breakthrough have greater uncertainties. For example, we have shown that deep cost reductions of geothermal power could motivate its widespread use in a least-cost system, which strongly motivates efforts to reduce the costs of geothermal electricity generation. However, if wind power also achieves substantial cost reductions, there would be less motivation to deploy geothermal power. And if electricity storage and solar costs were also to decline dramatically, even low-cost geothermal power might not be able to compete. Developers can engage in research and development (R&D) to try to reduce the costs of a particular technology, but that technology’s success is contingent on the cost of competing technologies. Building a large amount of geothermal power in anticipation of future cost reductions that do not materialize, can result in relatively high system costs.

These considerations suggest a strategy to deploy whatever clean technologies are economically viable today while engaging in actions that create and maintain a diverse set of options for the future, such as a research and development activities aimed at reducing tomorrow’s costs across a broad spectrum of energy technologies.

### Limitations of the study

In this study, we use a linear optimization electricity system model, the MEM, to examine the potential impact of applying different technology cost estimates on simulations of future decarbonized electricity systems. Instead of capturing the realistic market status or technical details, MEM is constructed in an idealized way to facilitate a fundamental understanding of electricity system dynamics in the context of carbon mitigation. The simplicity of MEM allows us to run hundreds or thousands of cases and examine a broad range of cost combination scenarios, which is difficult for models with complex structure and sophisticated underlying assumptions. The model can be easily extended to include more generation and storage technologies, constraints, and even change the objective function to fit other purposes. In this study, we isolate the impact of uncertain technology cost estimates in the future by changing only cost inputs, while keeping other factors unchanged, such as the model configuration, discount rate, hourly electricity demand, and wind and solar generation potential profiles. We provide an idealized framework and transparent analysis with full details disclosed. Our study does not serve to directly inform decision-making or design of the power system, but we hope to inspire future discussions and potential detailed works considering advanced models and sophisticated assumptions. Caveats should be kept in mind when interpreting results presented in this analysis.

Our quantitative results are contingent upon the assumed probability distribution of future technology costs. No one can generate probability distributions of future technology costs that will be universally accepted. To illustrate the potential influence of different technology cost assumptions, while avoiding our own appraisal of probability distributions of future costs, we use the NREL 2021 ATB estimated levels for advanced, moderate, and conservative cost innovation trajectories, and assign each of these trajectories with equal probability. Of course, these cost trajectories might not be equally likely achievable in today’s view. For example, the advanced technology innovation scenario pathway assumes substantial technology innovations and cost reductions associated with geothermal (e.g., substantial drilling advancements), which might need great policy support and investments. Also, the conservative technology innovation scenario assumes lower levels of R&D investment with minimal technology advancement and cost reduction for wind and solar, inconsistent with current trends. Future works using different weighting approaches are encouraged. Considering equal probability also enables us to examine the distribution of system responses under various technology cost levels.

Cost trajectories taken from the NREL 2021 ATB dataset do not cover the full range of future technology cost distribution potential. For example, three technologies (i.e., gas, nuclear, and biopower) in our analysis are assigned with only one future cost trajectory while three trajectories are considered for other technologies. A previous report has found that a nuclear power plant with an advanced reactor design could potentially reach a capital cost as low as $4000/kW,^58^ the impact of which can be partially represented by the low-cost geothermal trajectory. A recent work,^25^ based on the probabilistic cost forecasts approach, has shown that technologies such as solar, wind, and battery might reach lower cost levels than values from the advanced innovation pathways. To reflect such possibilities, we have conducted additional cases where costs for solar, wind, and battery are 30%, 50%, and 70% of the **LowCost** levels. These lower cost assumptions further enlarge benefits of the variable renewable technologies, leading to greater dispatches from these technologies and system cost reductions (Figures S18 and S19). However, as long as the high-cost projections stay possible for these technologies, qualitative conclusions from our analysis, such as that both variable renewables and firm technologies could dominate the electricity system, and that electricity systems with lower expected costs may have lower uncertainties in realized costs, remain robust. Regional differences in technology cost reduction potential, resource availability, and electricity demand^32^^,^^62^ would also influence optimization results. In addition, our least-cost solutions do not consider many practical issues faced by system planners. For example, environmental and social considerations may lead to the selection of one technology over another (e.g., land-use, visual and acoustic pollution, transmission rights-of-way, and safety concerns).

Many studies have incorporated the learning-by-doing process when modeling future energy system costs.^25^^,^^43^^,^^63^^,^^64^^,^^65^^,^^66^ Our analysis uses future technology cost estimates under various scenarios at annual time step taken directly from the NREL ATB dataset.^37^ These scenarios provided by the NREL ATB dataset are established based on assumptions including different learning rates, deployment scales, and innovation levels. Therefore, learning effect is implicitly considered but not explicitly modeled. It should be noted that projections based on experience curves embody substantial uncertainty,^25^^,^^67^ with ranges wider than that represented in this analysis. Such degree of uncertainty in the experience-curve-based cost projections likely precludes confident prediction of the composition of least-cost electricity systems for 2050. Meanwhile, learning by doing is not the only factor that determines future cost changes. Previous study has shown that the largest share of cost reduction in lithium-ion batteries was driven by public and private research and development^68^; another example would be the cost reduction potential for advanced nuclear plant mentioned above^58^; Cost saving innovations could lead to enhanced adoptions of new technologies that are currently expensive and immature.^66^ Our analysis is designed to isolate the impact of cost uncertainty and stay traceable for transparency. Future work should focus on better understanding how uncertainty in experience-curve-based cost projections translates into uncertainty in least-cost energy system transitions^65^^,^^69^^,^^70^^,^.

Our main cases represent least-cost optimizations considering conditions in a single year treated as a quasi-steady-state, with both fixed and variable costs prescribed. In the real world, it would take decades to build the electricity system, during which costs would evolve at different rates for different technologies.^71^^,^^72^^,^^73^ We conducted idealized transient simulations and found that in cases with gradually decreasing emission constraints, it could be costly to fully eliminate fossil fuels from the system. Transient simulations also end up with more wind and solar and less firm technologies at the year 2050, given the long capacity lifetime.

Our simulations use a constant discount rate of 7% in determining the cost of capital recovery. Preferred values for discount rates could vary among different regions and technologies.^74^ Higher discount rates tend to favor technologies such as gas, with lower fixed costs and higher variable costs. Since our discussions focus on deep emission reduction scenarios (e.g., 100% emission reduction), in which most technologies excluding biopower are dominated by capital costs rather than operating costs, changes in discount rate will have only minor impact on the cost ratio among technologies or our qualitative conclusions. For instance, ratios of cost between solar and geothermal under a 3% or 15% discount rate differ by less than 0.3% compared to the default discount rate case (7%) for all three cost trajectories.

Our analysis considers a limited number of technologies. There are other possible low-carbon emission choices that can provide additional values to the system.^22^^,^^23^^,^^47^^,^^75^ For example, we did not include long-duration storage in our main cases since its future cost projections are not directly available from the ATB dataset. Considering that adding additional flexibility on seasonal scale could potentially benefits variable renewables more than firm technologies, we have added simulations with long-duration storage represented by a hydrogen storage system, which has three separate parts in the model: the electrolyzer produces hydrogen (H_2_) using electricity, hydrogen storage stores the produced H_2_ underground, and the fuel cell converts H_2_ back to electricity. We scale the fixed cost of different components of the hydrogen storage system that represents a wide range of future cost reduction potentials. We also consider cases with an idealized representation of direct air capture of CO_2_. We add these technologies to the representative 2050 cost scenarios, respectively, and consider the 100% emission reduction constraint. Our results suggest that (Figure S20) low-cost hydrogen storage systems could benefit wind and solar by providing additional systematic flexibility with most benefits coming from the fuel cell component. In deeply decarbonized scenarios, low-cost hydrogen storage systems would also diminish the value of nuclear and geothermal. Reducing the cost of direct air capture enables fossil fuel generations under deep emission reduction constraints, and benefits wind and solar (Figure S21). As the cost of air capture approaches zero, reducing direct air capture costs in our model shifts the system to the one dominated by gas.

Our simulations use cost projections provided in the 2021 ATB dataset, which was updated later by NREL. A more recent version (2022 ATB dataset version 3, accessed in March, 2023) shows consistent cost trajectories for most technologies, with more cost reduction potential for geothermal under the advanced technology innovation trajectory (Figure S22). Taking into consideration the more recent numbers would make geothermal more competitive compared to other technologies when following its low-cost projection. Meanwhile, since the cost reduction potential for geothermal is modest under the conservative technology innovation, geothermal would remain uncompetitive when following its high-cost projection. Therefore, using the more recent ATB cost projections will not affect our qualitative conclusions.

In our main cases, we use the same electricity demand, originally derived from EIA,^76^ and combine it with concurrent generation profiles and different technology cost combinations. In reality, electrification of other sectors (e.g., transportation, industry, and construction) would change the shape and increase the variability of the demand profile (since we normalize the annual mean demand to 1 kWh, increases in magnitude of annual demand do not affect our conclusions here). To assess impact from a more variant electricity demand profile, we consider two additional scenarios: one with a demand profile calculated from the NREL Electrification Future Study (EFS) report^60^^,^^61^; and the other scenario using an idealized demand profile, which is calculated as the square of the default 2019 demand profile for each hour ($E_{i_{new}}=E_{i_{old}}^{2}$) from EIA. Both scenarios lead to slightly more use of variable renewables under the representative 2050 scenarios and larger system costs when approaching 100% emission reduction, while they do not change the qualitative conclusions of our analysis (Figure S23). Our approach still applies a static demand profile, which is not allowed to shift in time to reflect part of the advantage from electrification (e.g., demand management through electric vehicle charging). Allowing the demand to shift in time with low marginal cost is expected to promote renewables with cheaper battery storage and long-duration storage.

Many other considerations are important in making real-world decisions on investment strategy for a portfolio of technologies.^64^ Some of them are beyond the scope of this analysis and others are difficult to assess under our idealized framework. A few examples include that our analysis uses hourly time steps, whereas reliability of power systems can depend on grid events occurring on time scales of milliseconds to minutes.^77^ Similarly, we do not consider many other operational constraints that might be crucial to ensure the reliable operation of a realistic power system (e.g., ramping constraint of the nuclear power plant). There are uncertainties other than cost inputs when modeling energy systems, and these uncertain factors might interact with each other and produce unanticipated outcomes.^40^^,^^53^ The optimization of our least-cost systems depends heavily on given cost inputs and other assumptions (e.g., carbon emission constraint and capacity factor profiles), while in reality there are many other factors that would incentivize or disincentivize the deployment of certain technology. During the optimization, MEM has perfect foresight of future electricity needs and wind and solar resource availability, and assumes free and lossless conduction of electricity within the simulated region. These assumptions confer advantages on variable renewables relative to more realistic representations. Avoidance of fossil CO_2_ emissions would need to occur across all sectors to fully decarbonize the whole society. Some sectors that depend on high temperatures (e.g., steel, cement), high amounts of power (e.g., space heating), or high energy density (e.g., aviation) may prove more difficult to mitigate.^8^ Zero-emission technologies such as the concentrated solar power^24^ that can provide both electricity and thermal needs, and hydrogen that can be combusted to provide electricity, serve as long-term storage, and be used as liquid fuel might play important roles in future energy systems. Our model represents only the electricity sector as a single-node system, which does not consider the impacts of interconnections among multiple regions and the bridge between the electricity system and the non-electrified parts of the energy system.^78^^,^^79^

## STAR★Methods

### Key resources table

**Table:** 

REAGENT or RESOURCE	SOURCE	IDENTIFIER
**Deposited data**		

ERA5 weather records	European Centre for Medium-Range Weather Forecasts	https://cds.climate.copernicus.eu/cdsapp#!/dataset/reanalysis-era5-single-levels
Electricity demand	Ruggles et al. (2020)	https://www.nature.com/articles/s41597-020-0483-x
NREL cost estimates	National Renewable Energy Laboratory 2021 Annual Technology Baseline (ATB) report	https://atb.nrel.gov/electricity/2021/data
Lifecycle carbon emission estimates	Schlömer, S. et al. (2014)	https://www.ipcc.ch/site/assets/uploads/2018/02/ipcc_wg3_ar5_annex-iii.pdf
Data and code for this analysis	Zenodo	https://zenodo.org/records/10116066

**Software and algorithms**		

Macro Energy Model	GitHub and Zenodo	https://github.com/LDuan3008/MEM_CostUncertainty https://zenodo.org/records/10116066
Solar and wind capacity factor calculation	GitHub	https://github.com/carnegie/Create_Wind_and_Solar_Resource_Files
Gurobi v9.0	Gurobi Optimization	https://www.gurobi.com/
Python v3.7	Python Software Foundation	https://www.python.org/

### Resource availability

#### Lead contact

Further information and requests for resources should be directed to and will be fulfilled by the lead author, Lei Duan (leiduan@carnegiescience.edu).

#### Materials availability

This study did not generate new materials.

#### Data and code availability

• Data: Key model outputs that are used to support findings presented in this study have been deposited at Zenodo: https://zenodo.org/records/10116066. The original model outputs can be shared upon requests.
• Code: All original codes, including model codes and post-processing scripts, are written in Python and have been deposited at Zenodo with the same link above and at GitHub: https://github.com/LDuan3008/MEM_CostUncertainty. All codes are publicly available as of the date of publication.
• Any additional information required to reanalyze the data reported in this paper is available from the lead contact upon request.

### Method details

#### Model and simulations descriptions

In this study, we used the Macro Energy Model (MEM)^23^^,^^24^^,^^47^^,^^49^^,^^55^ that represents the electricity system as a linear-optimization problem under given input data, including technology costs, hourly electricity demand profile, and hourly generation potential for wind and solar. Complete model equations are provided in the section **Model formulation** below. The objective function of the model is to minimize total system cost. MEM considers techno-economic factors only, without including many real-world constraints or considerations that are important for a realistic power system (see discussion). MEM makes decisions about capacity and electricity dispatch based on relative costs (or ratios of cost) among technologies. There are no pre-existing capacities in the system in the main configuration of simulations. Since the demand and variable renewable resources are prescribed before optimizations, the model has perfect knowledge of forthcoming demand peaks or power shortages for renewable generations. Due to data availability and high computation intensity with increasing simulation length, here we conducted a series of simulations that last one calendar year using an hourly time resolution. Technologies used in this configuration of MEM include gas, gas with carbon capture and storage (gas-with-CCS), solar, onshore wind, offshore wind, nuclear, geothermal, biopower, and battery storage (Figure 1). We do not include coal because coal is more expensive and produces more carbon emissions than gas in the ATB dataset, and thus it will never compete with gas in the least-cost solution. Electricity inputs from the above technologies are used to meet demand, or have been curtailed at each hourly time step to maintain balance of the electricity exchange node at no cost. There is no lost load allowed during the simulations, and 100% of electricity demand is forced to be satisfied.

In current configuration of MEM, a fixed cost component is associated with all technologies to represent the fixed capital investment including the purchase and installation costs, and fixed operation & management (O&M) costs, and a variable cost is specified for gas, gas-with-CCS, nuclear, and biopower that includes variable O&M and fuel costs as appropriate (Table S1). For nuclear, we assume that the nuclear reactor must be operated at constant rates, and thus operation and fuel costs (i.e., variable costs) are added to fixed cost as dollar per unit capacity. The original cost assumptions for all generation and storage technologies, including the capital cost, fixed O&M expenses, variable operation and maintenance expenses, and fuel costs, are taken directly from the NREL 2021 ATB dataset.^37^ We then convert these cost values to fixed and variable cost components used in MEM considering the same discount rate (7% per year) in the same manners. Note that the solar cost taken directly from the NREL ATB dataset is in units of $/kW-AC and we do not consider factors such as the inverter loading. This might lead to slightly larger numbers as model inputs, while the difference is much smaller compared to model inputs under various cost projection trajectories.

The NREL ATB dataset provides cost estimates for different technologies for three cost reduction pathways covering periods from year 2019 to year 2050. These cost reduction scenarios are named Advanced, Moderate, and Conservative Technology Innovation pathways, and are defined based on different assumptions of learning rates, deployment scales, and innovation levels. Costs under these three pathways are more consistent in near-current times (i.e., same in year 2019) and diverge from each other with time, leading to substantially different cost projections by year 2050. Our analysis considers the year-2019 scenario to represent the near-current cost levels, and three year-2050 cost levels representative of Advanced, Moderate, and Conservative Technology Innovation pathways, corresponding to low, middle, and high future cost projections. We do not make our projections of future technology costs. For gas, nuclear, and biopower that have only one future cost scenario in the 2021 ATB dataset, we use that single cost level in our simulations. For offshore wind, we choose the Class 8 floating wind turbine technology, which is cheaper than the Class 12 wind turbine technology that represents NREL’s most recent assessment of the resource characteristics of mid-term deployment for floating technology in the California Call Areas defined by the Bureau of Ocean Energy Management (BOEM). For geothermal, we consider the flash enhanced geothermal system (EGS) to reflect larger geothermal resource potential in the United States. Such approach is used to reduce the overall numbers of ensemble members and to narrow down the scope of the analysis. The derived costs to deploy 1 kW nameplate capacity for solar and wind are cheaper compared to that for gas plant. However, gas remains as the cheaper option under zero emission reduction constraint due to renewables’ smaller capacity factors and higher variabilities. We do not pretend to know which cost projections (within or outside the range of NREL’s estimates) are more likely to happen in the future, and thus we treat each cost level equally during the simulations. MEM determines both capacity and electricity dispatch at each hourly time step based on ratios of technology costs, instead of the absolute cost values. That is, if all technologies experience the same percentage of cost reductions (both fixed and variable costs) in year-2050 compared to year-2019, the optimized technology capacities and hourly dispatches would be the same despite decreases in system cost.

In our analysis, we first run simulations under the year-2019 cost estimates, and then run four representative year-2050 cost combination cases: **HighCost**, where all technologies (excluding gas, nuclear, and biopower, each of which has only one future cost projection) follow the conservative technology innovation trajectories and achieve high future cost projections; **HighCost_LowGeo**, where only geothermal follows the advanced technology innovation trajectory and achieves the low cost projection, while other technologies remain at high cost projections; **HighCost_LowWindGeo**, where both wind and geothermal follow the advanced technology innovation trajectories and are at their low cost projection levels, while other technologies remain at high costs projections; and **LowCost**, where all technologies achieve the advanced innovation pathways and achieve their low cost projections. We run these simulations under multiple emission reduction constraints, in which CO_2_ emissions from fossil fuel-based generation sources (i.e., gas and gas-with-CCS) are gradually eliminated. Our analysis focuses on restricting CO_2_ emissions from gas and gas-with-CCS since they have much more direct carbon emissions than those of other low-carbon emission sources used here.^1^ The upper boundary limits of CO_2_ emissions from gas and gas-with-CCS vary from 100% to 0% with an increment of 2%, plus additional deeply decarbonized scenarios—99%, 99.9%, and 99.999%—relative to the case, in which all demands are met by gas. Changes in the Stack-plot would be smoother if a higher granularity in emission reduction constraint (i.e., < 2%) were used. We then perform year-2050 simulations considering all possible cost combinations under the 100% emission reduction constraint. Since there is no carbon removal approach in the main cases, the full decarbonization scenarios represent an “absolute zero” carbon emission condition (not accounting for cradle-to-gate emissions of generating technologies). To examine the potential temporal lock-in effect, we conduct idealized transient simulations with time-evolving emission reduction constraints and technology costs. The least-cost solution is found using the Gurobi optimizer written in Python.

#### Model formulations

We summarize the complete model formulation and nomenclature in this section.

In MEM, fixed costs of generation and storage technologies ($c_{fixed}$) are calculated as:

(Equation 1) $$\begin{array}{c} c_{fixed}=\frac{\gamma c_{capital}+c_{fixed\_O\&M}}{h} \end{array}$$

Where $c_{capital}$ is the capital cost in units of $/kW for generation technologies and $/kWh for storage technology, $c_{fixed\_O\&M}$ is the fixed operating and maintenance (O&M) cost in units of ($/year)/kW for generation technologies and ($/year)/kWh for storage technology, h is the number of hours per year, and γ represents the capital recovery factor that is calculated based on discount rate (i, unitless) and technology lifetime (n, in units of years):

(Equation 2) $$\begin{array}{c} \gamma =\frac{i{\left(1+i\right)}^{n}}{{\left(1+i\right)}^{n}-1} \end{array}$$

Variable cost for technologies such as gas, gas-with-CCS and biopower ($c_{var}$) is calculated as the sum of fuel costs ($c_{fuel}$) and variable O&M cost ($c_{var\_O\&M}$).

Along with cost inputs, a number of constraints are applied to different technologies to ensure that reasonable optimization results can be found. These constraints cover technology parameters such as technology capacity, dispatch, and energy stored in storage. For example, for technology capacity of generation ($C^{g}$) and storage ($C^{s}$) technologies:

(Equation 3) $$\begin{array}{c} 0\leq C \end{array}$$

For dispatch from generations at time step t ($D_{t}^{g}$):

(Equation 4) $$\begin{array}{c} 0\leq D_{t}^{g}\leq C^{g}f^{g} \end{array}$$

Where $f^{g}$ is unitless and represents the capacity factor for various technologies (time series with the same length as time steps and values between 0 and 1 for solar, onshore wind, and offshore wind, and constant 1 for other technologies).

Dispatch to battery storage ($D_{t}^{to\_S}$, charging) and from battery storage ($D_{t}^{from\_S}$, discharging) are constrained by both the storage capacity ($C^{s}$) and storage charging duration ($\tau^{s}$):

(Equation 5) $$\begin{array}{c} 0\leq D_{t}^{to\_S}\leq \frac{C^{S}}{\tau^{s}} \end{array}$$

And

(Equation 6) $$\begin{array}{c} 0\leq D_{t}^{from\_S}\leq \frac{C^{S}}{\tau^{s}} \end{array}$$

Discharge from battery storage is also constrained by the total amount of energy stored in storage at time step t ($S_{t}$):

(Equation 7) $$\begin{array}{c} 0\leq D_{t}^{from\_S}\leq S_{t}(1-\delta^{s}) \end{array}$$

Where $\delta^{s}$ is in units of 1/hour and is the storage decay rate or energy loss per hour expressed as fraction of energy in storage, and $S_{t}$ satisfies:

(Equation 8) $$\begin{array}{c} 0\leq S_{t}\leq C^{S} \end{array}$$

Storage energy balance at any time step t+1 is calculated as:

(Equation 9) $$\begin{array}{c} S_{t+1}=S_{t}\left(1-\delta^{s}\right)+\eta^{S}D_{t}^{to\_S}-D_{t}^{from\_S} \end{array}$$

And

(Equation 10) $$\begin{array}{c} S_{1}=S_{t_{end}}\left(1-\delta^{s}\right)+\eta^{S}D_{1}^{to\_S}-D_{1}^{from\_S} \end{array}$$

Where $\eta^{S}$ is a unitless variable representing the storage round-trip efficiency.

At any point, the system energy balance requirement should be satisfied. That is, the sum of energy inflow to the exchange node should equal the sum of energy outflow from the exchange node. This is represented in the model by:

(Equation 11) $$\begin{array}{c} \sum_{g}D_{t}^{g}+D_{t}^{from\_S}=M_{t}+D_{t}^{to\_S}+Curtail \end{array}$$

$M_{t}$ is the electricity demand at time step t and Curtail is the curtailed energy that is not used or charged to storage. The objection function is designed to minimize total system cost, which is calculated as:

(Equation 12) $$\begin{array}{c} \sum_{g}c_{fixed}^{g}C^{g}+\sum_{s}c_{fixed}^{s}C^{s}+\sum_{g}\frac{\sum_{t}c_{var}^{g}D_{t}^{g}}{h} \end{array}$$

For long-duration storage, we have used three separate components to represent the power-to-gas-to-power (PGP) system. to_PGP and from_PGP convert energy into different forms and then transfer energy between nodes. The dispatch from to_PGP ($D_{t}^{to\_PGP}$) and from_PGP ($D_{t}^{from\_PGP}$) is constrained as:

(Equation 13) $$\begin{array}{c} 0\leq D_{t}^{to\_PGP}\leq C^{to\_PGP} \end{array}$$

(Equation 14) $$\begin{array}{c} 0\leq C^{to\_PGP} \end{array}$$

And

(Equation 15) $$\begin{array}{c} 0\leq D_{t}^{from\_PGP}\leq C^{from\_PGP} \end{array}$$

(Equation 16) $$\begin{array}{c} 0\leq C^{from\_PGP} \end{array}$$

PGP storage behaves similarly to battery storage. Energy stored in PGP storage at time step t+1 (${S_{PGP}}_{t+1}$) is calculated as:

(Equation 17) $$\begin{array}{c} {S_{PGP}}_{t+1}={S_{PGP}}_{t}\left(1-\delta^{PGP}\right)+\eta^{to\_PGP}D_{t}^{to\_PGP}-D_{t}^{from\_PGP} \end{array}$$

And

(Equation 18) $$\begin{array}{c} {S_{PGP}}_{1}={S_{PGP}}_{t_{end}}\left(1-\delta^{PGP}\right)+\eta^{to\_PGP}D_{1}^{to\_PGP}-D_{1}^{from\_PGP} \end{array}$$

Where $\eta^{to\_PGP}$ represents efficiency that to_PGP transfers energy from the main node to PGP storage. Energy transferred from PGP storage to the main node through from_PGP is calculated as:

(Equation 19) $$\begin{array}{c} \eta^{from\_PGP}D_{t}^{from\_PGP} \end{array}$$

With $\eta^{from\_PGP}$ being efficiency of from_PGP. Correspondingly, the system balance equation becomes:

(Equation 20) $$\begin{array}{c} \sum_{g}D_{t}^{g}+D_{t}^{from\_S}+\eta^{from\_PGP}D_{t}^{from\_PGP}=M_{t}+D_{t}^{to\_S}+D_{t}^{to\_PGP}+Curtail \end{array}$$

#### Demand and resource data

In our simulations, we combine different cost inputs with the same hourly demand and variable renewable generation potential profiles to find the least-cost solutions of electricity systems. We provide the model with concurrent hourly electricity demand and variable renewable potential data from year-2016 to year-2019. The central cases show results with the year-2019 inputs, and others are plotted in the Supplementary Information. Hourly demand data were originally collected from the U.S. Energy Information Administration (EIA), which covers all the contiguous United States. We normalize the demand profile before simulations by dividing the annual mean demand on each hourly step so that mean demand equals 1 kWh. A cleaning approach is then applied to create the final complete and usable data records used in this analysis.^76^

Hourly wind and solar capacity factors are estimated using the Medium-Range Weather Forecasts (ECMWF) ERA5^80^ dataset, which has a resolution of 0.25° by latitude and 0.25° by longitude. For wind, we assume wind turbines with a hub height of 100 m and use the wind speed at the corresponding height provided by the dataset. A piecewise function is used to calculate wind capacity factor^31^^,^^81^^,^^82^^,^^83^: (i) below a cut-in speed ($u_{ci}$) of 3 m s^-1^ the capacity factor is zero, (ii) between the cut-in speed of 3 m s^-1^ and rated speed ($u_{r}$) of 12 m s^-1^ the capacity factor is ${u_{ci}}^{3}/{u_{r}}^{3}$, (iii) between the rated speed of 12 m s^-1^ and the cut-out speed ($u_{co}$) of 25 m s^-1^ the capacity factor is 1.0, and (iv) above the cut-out speed of 25 m s^-1^ the capacity factor is zero. We assume a single-axis tracking solar panel system with north-south direction, with a tilt of 0° and a maximum tuning angle of 45°. Solar zenith angle and incidence angle are calculated based on geographic location and local time. The in-panel solar radiation is then calculated, separating direct and diffuse radiation components based on an empirical piecewise model in combination with the surrounding panel temperature, to calculate hourly averaged solar capacity factors.^84^^,^^85^^,^^86^ A GitHub repository (https://github.com/carnegie/Create_Wind_and_Solar_Resource_Files) is created to demonstrate how raw ERA5 weather data can be converted into capacity factors.

We first calculate both wind and solar capacity factors at each grid cell at the same resolution of the original ERA5 product, and then integrate grid cells by calculating the area-weighted mean values to generate continental-scale hourly profiles. For onshore wind and solar, our central scenarios consider cases in which only the top 25% grid cells in the continental U.S. that have the largest capacity factors are aggregated by doing an area-weighted average calculation. For the offshore wind, we consider the top 25% ocean grids of the U.S. Exclusive Economic Zone (EEZ) that have the largest capacity factors. The resulting annual mean capacity factors across different years are about 0.26, 0.29, and 0.52 for solar, wind, and offshore wind, respectively. Our capacity factor calculations represent only a first order estimate of the spatial and temporal characteristics of wind and solar resources. Using a profile with better wind and solar resource potential (e.g., larger annual mean capacity factor and/or less variations) is numerically equivalent to having lower technology costs (relative to other technologies) in our optimization framework.

### Quantification and statistical analysis

Box and whisker plots such as Figure 4 that show the median and quartiles of the distribution of ensemble data are plotted using the Python package Matplotlib and the function *boxplot* (https://matplotlib.org/stable/api/_as_gen/matplotlib.pyplot.boxplot.html). The distribution plots such as Figure 5 that represent the probability density function of ensemble data are done using the Python package SciPy with a Gaussian kernel density estimation function *gaussian_kde* (https://docs.scipy.org/doc/scipy/reference/generated/scipy.stats.gaussian_kde.html). Ensemble mean and standard deviation are estimated with the Python package Numpy and the *numpy.mean* (https://numpy.org/doc/stable/reference/generated/numpy.mean.html) and *numpy.std* (https://numpy.org/doc/stable/reference/generated/numpy.std.html) functions.

## References

[1] Schlömer S., Bruckner T., Fulton L., Hertwich E., McKinnon A., Perczyk D., Roy J., Schaeffer R., Sims R., Smith P. (2014). Climate Change 2014: Mitigation of Climate Change: Contribution of Working Group III to the Fifth Assessment Report of the Intergovernmental Panel on Climate Change.

[2] Rogelj J., Shindell D., Jiang K., Fifita S., Forster P., Ginzburg V., Handa C., Kheshgi H., Kobayashi S., Kriegler E. (2018). Global Warming of 1.5°C. An IPCC Special Report on the impacts of global warming of 1.5°C above pre-industrial levels and related global greenhouse gas emission pathways, in The Context of Strengthening the Global Response to the Threat of Climate Change, Sustainable Development, and Efforts to Eradicate Poverty (Intergovernmental Panel on Climate Change).

[3] Krey V., Luderer G., Clarke L., Kriegler E. (2014). Getting from here to there – energy technology transformation pathways in the EMF27 scenarios. Clim. Change.

[4] Kriegler E., Weyant J.P., Blanford G.J., Krey V., Clarke L., Edmonds J., Fawcett A., Luderer G., Riahi K., Richels R. (2014). The role of technology for achieving climate policy objectives: overview of the EMF 27 study on global technology and climate policy strategies. Clim. Change.

[5] Steinberg D., Bielen D., Eichman J., Eurek K., Logan J., Mai T., McMillan C., Parker A., Vimmerstedt L., Wilson E. (2017).

[6] Leibowicz B.D., Lanham C.M., Brozynski M.T., Vázquez-Canteli J.R., Castejón N.C., Nagy Z. (2018). Optimal decarbonization pathways for urban residential building energy services. Appl. Energy.

[7] Luderer G., Vrontisi Z., Bertram C., Edelenbosch O.Y., Pietzcker R.C., Rogelj J., De Boer H.S., Drouet L., Emmerling J., Fricko O. (2018). Residual fossil CO2 emissions in 1.5–2 °C pathways. Nat. Clim. Change.

[8] Davis S.J., Lewis N.S., Shaner M., Aggarwal S., Arent D., Azevedo I.L., Benson S.M., Bradley T., Brouwer J., Chiang Y.-M. (2018). Net-zero emissions energy systems. Science.

[9] Luderer G., Madeddu S., Merfort L., Ueckerdt F., Pehl M., Pietzcker R., Rottoli M., Schreyer F., Bauer N., Baumstark L. (2021). Impact of declining renewable energy costs on electrification in low-emission scenarios. Nat. Energy.

[10] Yang X.J., Hu H., Tan T., Li J. (2016). China’s renewable energy goals by 2050. Environmental Development.

[11] De León K. (2018).

[12] Hmg (2019).

[13] Ocasio-Cortez A., Markey S.E. (2019). 116th Congress, 1st Session, H. Res.

[14] Meinshausen M., Lewis J., McGlade C., Gütschow J., Nicholls Z., Burdon R., Cozzi L., Hackmann B. (2022). Realization of Paris Agreement pledges may limit warming just below 2 °C. Nature.

[15] Brick S., Thernstrom S. (2016). Renewables and decarbonization: Studies of California, Wisconsin and Germany. Electr. J..

[16] Heal G. (2017). Reflections—What Would It Take to Reduce U.S. Greenhouse Gas Emissions 80 Percent by 2050?. Rev. Environ. Econ. Pol..

[17] Jenkins J.D., Zhou Z., Ponciroli R., Vilim R.B., Ganda F., de Sisternes F., Botterud A. (2018). The benefits of nuclear flexibility in power system operations with renewable energy. Appl. Energy.

[18] Sepulveda N.A., Jenkins J.D., de Sisternes F.J., Lester R.K. (2018). The Role of Firm Low-Carbon Electricity Resources in Deep Decarbonization of Power Generation. Joule.

[19] Gielen D., Boshell F., Saygin D., Bazilian M.D., Wagner N., Gorini R. (2019). The role of renewable energy in the global energy transformation. Energy Strategy Rev..

[20] Tapia-Ahumada K.D., Reilly J., Yuan M., Strzepek K. (2019).

[21] Yuan M., Tong F., Duan L., Dowling J.A., Davis S.J., Lewis N.S., Caldeira K. (2020). Would firm generators facilitate or deter variable renewable energy in a carbon-free electricity system?. Appl. Energy.

[22] Bistline J.E.T., Blanford G.J. (2021). Impact of carbon dioxide removal technologies on deep decarbonization of the electric power sector. Nat. Commun..

[23] Duan L., Petroski R., Wood L., Caldeira K. (2022). Stylized least-cost analysis of flexible nuclear power in deeply decarbonized electricity systems considering wind and solar resources worldwide. Nat. Energy.

[24] Kennedy K.M., Ruggles T.H., Rinaldi K., Dowling J.A., Duan L., Caldeira K., Lewis N.S. (2022). The role of concentrated solar power with thermal energy storage in least-cost highly reliable electricity systems fully powered by variable renewable energy. Advances in Applied Energy.

[25] Way R., Ives M.C., Mealy P., Farmer J.D. (2022). Empirically grounded technology forecasts and the energy transition. Joule.

[26] Newell R., Raimi D., Aldana G. (2019).

[27] IRENA (2020).

[28] Victoria M., Haegel N., Peters I.M., Sinton R., Jäger-Waldau A., del Cañizo C., Breyer C., Stocks M., Blakers A., Kaizuka I. (2021). Solar photovoltaics is ready to power a sustainable future. Joule.

[29] Jenkins J.D., Luke M., Thernstrom S. (2018). Getting to Zero Carbon Emissions in the Electric Power Sector. Joule.

[30] Antonini E.G.A., Ruggles T.H., Farnham D.J., Caldeira K. (2022). The quantity-quality transition in the value of expanding wind and solar power generation. iScience.

[31] Shaner M.R., Davis S.J., Lewis N.S., Caldeira K. (2018). Geophysical constraints on the reliability of solar and wind power in the United States. Energy Environ. Sci..

[32] Tong D., Farnham D.J., Duan L., Zhang Q., Lewis N.S., Caldeira K., Davis S.J. (2021). Geophysical constraints on the reliability of solar and wind power worldwide. Nat. Commun..

[33] Duan L., Ruggles T.H., Caldeira K. (2022). Electricity systems in the limit of free solar photovoltaics and continent-scale transmission. iScience.

[34] Wiliarty S.E. (2013). Nuclear Power in Germany and France. Polity.

[35] Morgan M.G., Abdulla A., Ford M.J., Rath M. (2018). US nuclear power: The vanishing low-carbon wedge. Proc. Natl. Acad. Sci. USA.

[36] Bouckaert S., Pales A.F., McGlade C., Remme U. (2021).

[37] Vimmerstedt L., Akar S., Mirletz B., Stright D., Augustine C., Beiter P., Cohen S., Cole W., Duffy P., Feldman D. (2021).

[38] (2022). Annual Energy Outlook - U.S. Energy Informbation Administration (EIA).

[39] Schlachtberger D.P., Brown T., Schäfer M., Schramm S., Greiner M. (2018). Cost optimal scenarios of a future highly renewable European electricity system: Exploring the influence of weather data, cost parameters and policy constraints. Energy.

[40] Pilpola S., Lund P.D. (2020). Analyzing the effects of uncertainties on the modelling of low-carbon energy system pathways. Energy.

[41] Shirizadeh B., Perrier Q., Quirion P. (2022). How sensitive are optimal fully renewable power systems to technology cost uncertainty?. Energy J..

[42] Hirth L., Steckel J.C. (2016). The role of capital costs in decarbonizing the electricity sector. Environ. Res. Lett..

[43] Heuberger C.F., Rubin E.S., Staffell I., Shah N., Mac Dowell N. (2017). Power capacity expansion planning considering endogenous technology cost learning. Appl. Energy.

[44] Bistline J.E., Blanford G.J. (2020). Value of technology in the U.S. electric power sector: Impacts of full portfolios and technological change on the costs of meeting decarbonization goals. Energy Econ..

[45] Phadke A., Paliwal U., Abhyankar N., McNair T., Paulos B., O’Connell R. (2020).

[46] Victoria M., Zhu K., Brown T., Andresen G.B., Greiner M. (2020). Early decarbonisation of the European energy system pays off. Nat. Commun..

[47] Dowling J.A., Rinaldi K.Z., Ruggles T.H., Davis S.J., Yuan M., Tong F., Lewis N.S., Caldeira K. (2020). Role of Long-Duration Energy Storage in Variable Renewable Electricity Systems. Joule.

[48] He G., Lin J., Sifuentes F., Liu X., Abhyankar N., Phadke A. (2020). Rapid cost decrease of renewables and storage accelerates the decarbonization of China’s power system. Nat. Commun..

[49] Ruggles T.H., Dowling J.A., Lewis N.S., Caldeira K. (2021). Opportunities for flexible electricity loads such as hydrogen production from curtailed generation. Advances in Applied Energy.

[50] Moret S., Codina Gironès V., Bierlaire M., Maréchal F. (2017). Characterization of input uncertainties in strategic energy planning models. Appl. Energy.

[51] Mavromatidis G., Orehounig K., Carmeliet J. (2018). A review of uncertainty characterisation approaches for the optimal design of distributed energy systems. Renew. Sustain. Energy Rev..

[52] Lopion, Robinius, Markewitz, Stolten, Robinius (2019). Cost uncertainties in energy system optimization models: A quadratic programming approach for avoiding penny switching effects. Energies.

[53] Scott I.J., Carvalho P.M., Botterud A., Silva C.A. (2021). Long-term uncertainties in generation expansion planning: Implications for electricity market modelling and policy. Energy.

[54] Neumann F., Brown T. (2023). Broad ranges of investment configurations for renewable power systems, robust to cost uncertainty and near-optimality. iScience.

[55] Henry C.L., Eshraghi H., Lugovoy O., Waite M.B., DeCarolis J.F., Farnham D.J., Ruggles T.H., Peer R.A., Wu Y., de Queiroz A. (2021). Promoting reproducibility and increased collaboration in electric sector capacity expansion models with community benchmarking and intercomparison efforts. Appl. Energy.

[56] Clack C.T.M., Qvist S.A., Apt J., Bazilian M., Brandt A.R., Caldeira K., Davis S.J., Diakov V., Handschy M.A., Hines P.D.H. (2017). Evaluation of a proposal for reliable low-cost grid power with 100% wind, water, and solar. Proc. Natl. Acad. Sci. USA.

[57] Denholm P., Arent D.J., Baldwin S.F., Bilello D.E., Brinkman G.L., Cochran J.M., Cole W.J., Frew B., Gevorgian V., Heeter J. (2021). The challenges of achieving a 100% renewable electricity system in the United States. Joule.

[58] (2018). Exploring the Role of Advanced Nuclear in Future Energy Markets: Economic Drivers, Barriers, and Impacts in the United States.

[59] Ziegler M.S., Trancik J.E. (2021). Re-examining rates of lithium-ion battery technology improvement and cost decline. Energy Environ. Sci..

[60] Sun Y., Jadun P., Nelson B., Muratori M., Murphy C., Logan J., Mai T. (2020).

[61] Zhou E., Mai T. (2021).

[62] Siala K., Mahfouz M.Y. (2019). Impact of the choice of regions on energy system models. Energy Strategy Rev..

[63] Nemet G.F. (2006). Beyond the learning curve: factors influencing cost reductions in photovoltaics. Energy Pol..

[64] Way R., Lafond F., Lillo F., Panchenko V., Farmer J.D. (2019). Wright meets Markowitz: How standard portfolio theory changes when assets are technologies following experience curves. J. Econ. Dynam. Control.

[65] Yao Y., Xu J.-H., Sun D.-Q. (2021). Untangling global levelised cost of electricity based on multi-factor learning curve for renewable energy: Wind, solar, geothermal, hydropower and bioenergy. J. Clean. Prod..

[66] Caldeira K., Duan L., Moreno-Cruz J. (2023). The value of reducing the Green Premium: cost-saving innovation, emissions abatement, and climate goals. Environ. Res. Lett..

[67] Lafond F., Bailey A.G., Bakker J.D., Rebois D., Zadourian R., McSharry P., Farmer J.D. (2018). How well do experience curves predict technological progress? A method for making distributional forecasts. Technol. Forecast. Soc. Change.

[68] Ziegler M.S., Song J., Trancik J.E. (2021). Determinants of lithium-ion battery technology cost decline. Energy Environ. Sci..

[69] Savage T., Davis A., Fischhoff B., Morgan M.G. (2021). A strategy to improve expert technology forecasts. Proc. Natl. Acad. Sci. USA.

[70] Kanyako F., Baker E., Anthoff D. (2023). Identifying low-carbon energy R&D portfolios that are robust when models and experts disagree. Joule.

[71] International Renewable Energy Agency IRENA (2019).

[72] International Renewable Energy Agency IRENA (2019).

[73] Schmidt O., Melchior S., Hawkes A., Staffell I. (2019). Projecting the Future Levelized Cost of Electricity Storage Technologies. Joule.

[74] Steffen B. (2020). Estimating the cost of capital for renewable energy projects. Energy Econ..

[75] Dioha M.O., Duan L., Ruggles T.H., Bellocchi S., Caldeira K. (2022). Exploring the role of electric vehicles in Africa’s energy transition: A Nigerian case study. iScience.

[76] Ruggles T.H., Farnham D.J., Tong D., Caldeira K. (2020). Developing reliable hourly electricity demand data through screening and imputation. Sci. Data.

[77] Anvari M., Lohmann G., Wächter M., Milan P., Lorenz E., Heinemann D., Tabar M.R.R., Peinke J. (2016). Short term fluctuations of wind and solar power systems. New J. Phys..

[78] Pickering B., Lombardi F., Pfenninger S. (2022). Diversity of options to eliminate fossil fuels and reach carbon neutrality across the entire European energy system. Joule.

[79] Tröndle T., Lilliestam J., Marelli S., Pfenninger S. (2020). Trade-Offs between Geographic Scale, Cost, and Infrastructure Requirements for Fully Renewable Electricity in Europe. Joule.

[80] Hersbach H., Bell B., Berrisford P., Hirahara S., Horányi A., Muñoz-Sabater J., Nicolas J., Peubey C., Radu R., Schepers D. (2020). The ERA5 global reanalysis. Q. J. R. Meteorol. Soc..

[81] Bett P.E., Thornton H.E. (2016). The climatological relationships between wind and solar energy supply in Britain. Renew. Energy.

[82] Clack C.T.M., Alexander A., Choukulkar A., MacDonald A.E. (2016). Demonstrating the effect of vertical and directional shear for resource mapping of wind power. Wind Energy.

[83] Sedaghat A., Hassanzadeh A., Jamali J., Mostafaeipour A., Chen W.-H. (2017). Determination of rated wind speed for maximum annual energy production of variable speed wind turbines. Appl. Energy.

[84] Huld T., Gottschalg R., Beyer H.G., Topič M. (2010). Mapping the performance of PV modules, effects of module type and data averaging. Sol. Energy.

[85] Pfenninger S., Staffell I. (2016). Long-term patterns of European PV output using 30 years of validated hourly reanalysis and satellite data. Energy.

[86] Reindl D.T., Beckman W.A., Duffie J.A. (1990). Diffuse fraction correlations. Sol. Energy.

