# Supplementary material for: Implications of uncertainty in technology cost projections for least-cost decarbonized electricity systems
Source: iScience. 2023 Dec 7;27(1):108685. doi: 10.1016/j.isci.2023.108685 (PMC10753084; doi:10.1016/j.isci.2023.108685)
Supplement: Document S1. Figures S1–S24, Tables S1–S3, and Text S1 and S2 [file mmc1.pdf]

iScience, Volume *27*

Supplemental information

Implications of uncertainty in technology cost projections for least-cost decarbonized electricity systems

Lei Duan and Ken Caldeira

This file contains:

Supplementary Text S1 to S2

Supplementary Figure S1 to S24

Supplementary Table S1 to S3

Supplementary Text S1. Results considering year-2019 cost levels.

In our simulations using the year-2019 NREL ATB cost levels, gas is the lowest-cost way to meet the electricity demand when there is zero emission reduction constraint. As emission constraints tighten, dispatch of electricity generation from gas is replaced with the two next least-costly options. These gas-with-CCS because it emits less carbon per unit electricity generated, and solar because it correlates with daily peak demand (Fig. S1). Further increases in emission reduction constraints limit the use of fossil fuels and lead to more costly wind and solar generation, with increased curtailment and flexible fossil fuel generations filling gaps between variable electricity supply and electricity demand (Fig. S1 and S2). Under very deep emission reduction constraints (e.g., > 90% emission reduction constraint), cost-effective gas, even with CCS, is carbon-constrained. This means it cannot be used to cost-effectively compensate for gaps in wind and solar generation, and the cost of batteries is high at year-2019 level. (Our model configuration does not consider direct air capture.) Such constraints promote use of nuclear power and biopower. Under the year-2019 NREL ATB cost estimates, the least-cost system is consisting of nuclear power providing constant and stable electricity generation, and wind and solar, with support from flexible biopower and battery storage to address variability in electricity generation and demand (Fig. S2). For the year-2019 cost scenario, gas-with-CCS dominates in a 60% emission reduction case, primarily due to the low variable costs for gas-with-CCS from the NREL 2021 ATB report. Additional cases, using a higher variable cost for gas and gas-with-CCS technologies[1], show more competitiveness of renewables under low emission reduction constraints (Fig. S24). Considering the lifecycle emissions, as in our default cases, leads to a lower CO_2 capture rate for gas-with-CCS (65%) due to infrastructure and supply chain emissions, and associated methane emissions. Instead, considering a higher CO_2 capture rate (e.g., 90% of emissions from gas are captured and stored) will allow gas-with-CCS to play a more important role under low to middle decarbonization constraints, but relatively less impact under deep emission reduction scenarios such as 99 and 100% (Fig. S24).

Supplementary Text S2. Stylized transient simulations.

Here we conduct stylized transient simulations starting from 2019 to 2050. In these simulations, emission constraint is prescribed to increase linearly from zero at 2019 to 100% at 2050, and cost inputs for different technologies are updated each year based on the NREL 2021 ATB report. We represent the effect of long-lasting capacity by keeping a fraction of technology capacities simulated from the previous year starting from the year 2020:

$$C_{fixed,n} = C_{total,n-1} * f$$

where f is calculated based on the technology lifetime ($f = 1 - 1/lifetime$) and $C_{total,n-1}$ is the total technology capacity from the previous year. The total technology capacity in any given year is the sum of the capacity from the previous year and the newly built capacity as a consequence of emission and cost reductions. This formulation considers built capacities to represent a population of power plants that can be treated statistically as a continuous quantity. The new capacity is built to optimize costs in the current year, with no foresight regarding needs in future years. Since the emission reduction constraint is enhanced yearly, there are increasingly fewer opportunities for low-emission generation technologies to compete under the updated cost inputs.

During the early years of the transient simulations, with modest emissions reduction constraints, with newly built and existing fossil fuel generation available to firm up electricity supply, wind and solar efficiently substitute for fuel use and provide a lower cost approach to meeting demand. In our analysis, all 729 ensemble members of transient simulations show newly built gas and gas-with-CCS capacities, which will last for decades until the end of simulations. As a result, for all transient cases there are fossil fuel capacities that remain in the system with no use at all under the 100% emission reduction constraints.

Compared to the single-year-2050 optimization results, simulated transient-year-2050 results show a somewhat higher system cost and a wider distribution of system costs across the 729 technology cost combinations (Fig. S14 and S15). The lowest system cost among the 729 least-cost solutions for transient-year-2050 scenario is \$0.07/kWh, ~ 14% larger than the lowest system cost for the single-year-2050 optimizations. The least-cost system with the highest system cost among the 729 ensemble members for transient-year-2050 scenario is \$0.11/kWh, 20% larger than that for the single-year-2050 optimizations. The spread of costs is therefore

larger in the transient case. In general, in the transient-year-2050 case relative to the single-year-2050 case, there is more wind, solar, offshore wind, and battery storage, and less geothermal. Once wind and solar capacity is built, they can produce effectively free electricity in the optimization process and thereby reduce capacities of firm technologies (mostly geothermal) (Fig. S16). Transient simulations shown here are highly idealized and results should be carefully interpreted.

Supplementary Figure S1. Contributions to total system costs, related to STAR Methods. Year-2019 cost levels are used in combination with year-2019 hourly electricity demand and renewable generation potential data (i.e., capacity factors). Similar to Figure 2, various emission reduction constraints are compared.

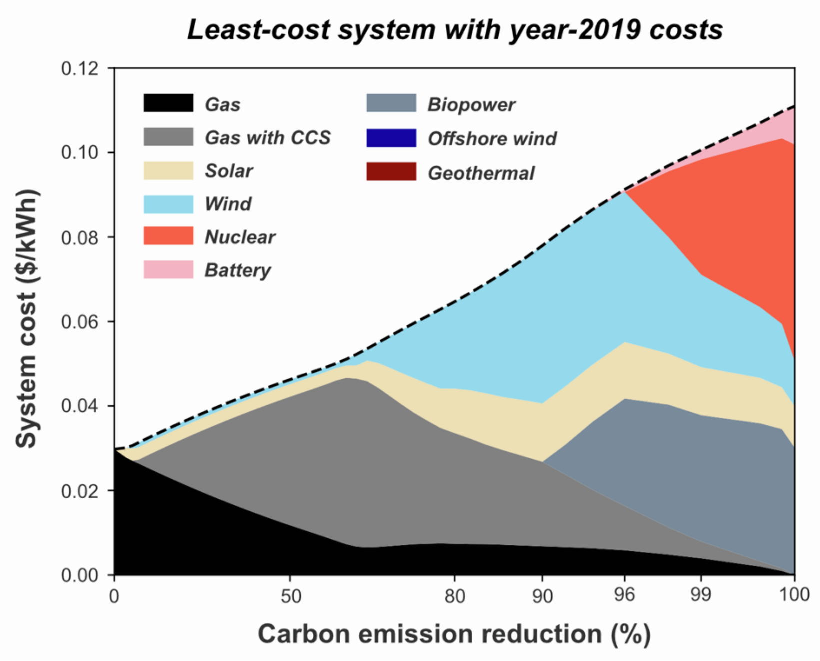

Supplementary Figure S2. Daily and hourly electricity dispatch, related to STAR Methods. Different emission reduction constraints (i.e., **A** 10%, **B** 20%, **C** 50%, **D** 80%, **E** 90%, **F** 96%, **G** 99%, and **H** 100%) are compared for cases using the year-2019 cost estimates.

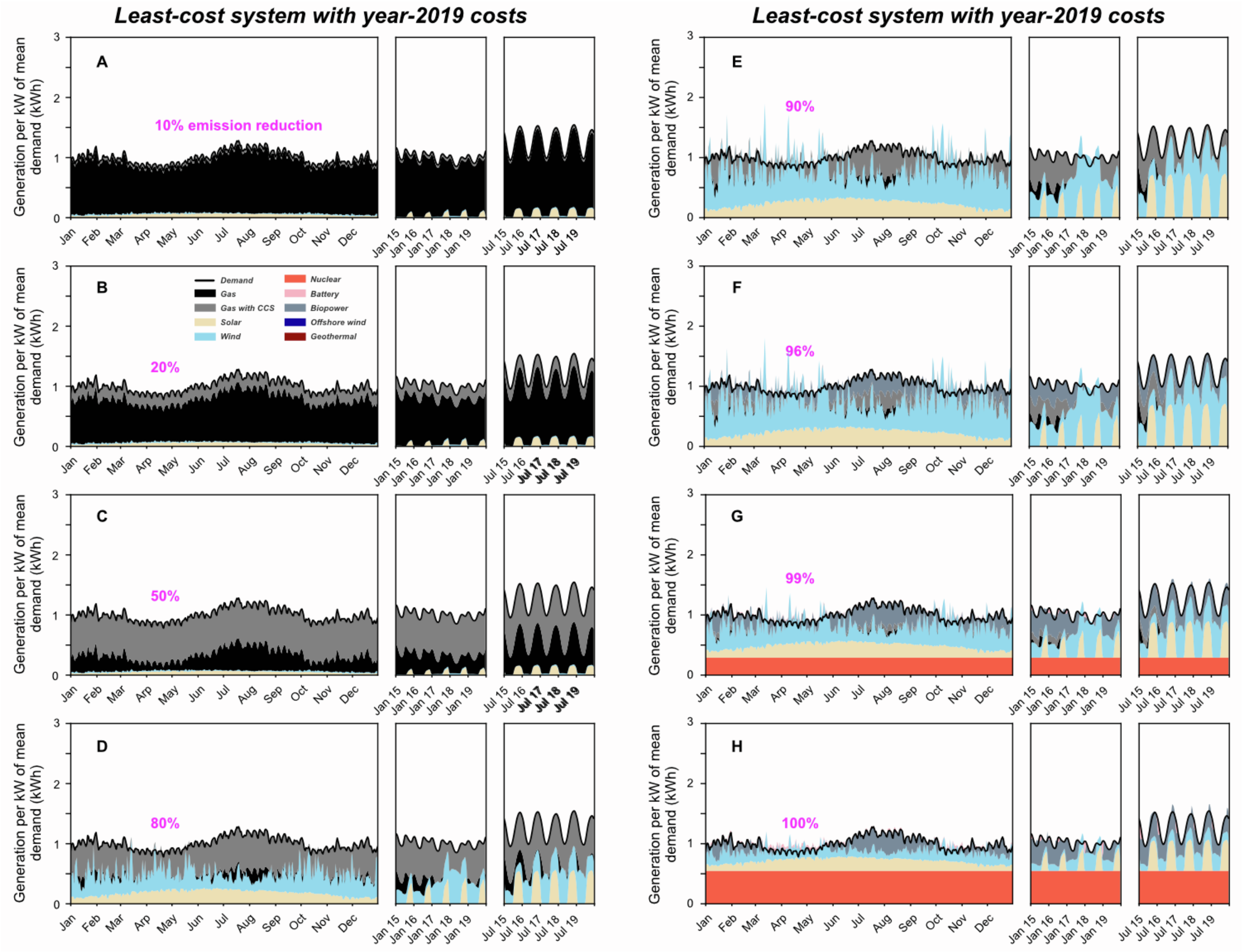

Supplementary Figure S3. Contributions to total system costs without biopower or nuclear, related to STAR Methods. Compared to a case that uses the full technology mixture (Fig. **S1**), (**A**) biopower is first removed from the system and then (**B**) both biopower and nuclear are removed.

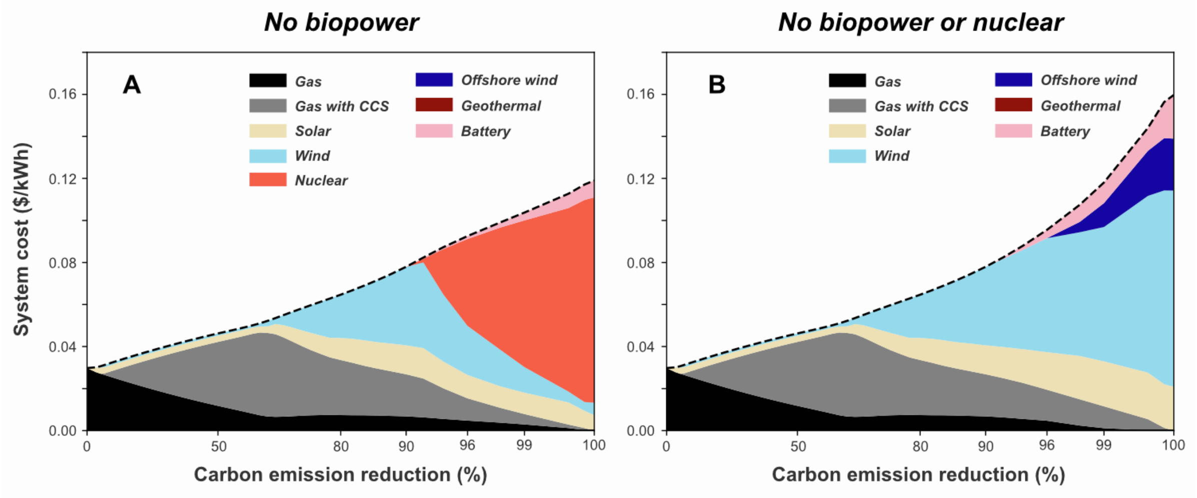

Supplementary Figure S4. Sensitivity analysis to different years' demand and generation potential inputs, related to STAR Methods. (**A** to **O**) Year 2016 to year 2018 hourly electricity demand and generation potential are combined with the year-2019 and four representative year-2050 cost combination cases. Our main cases (**P** to **T**) use the year 2019 demand and renewable potential.

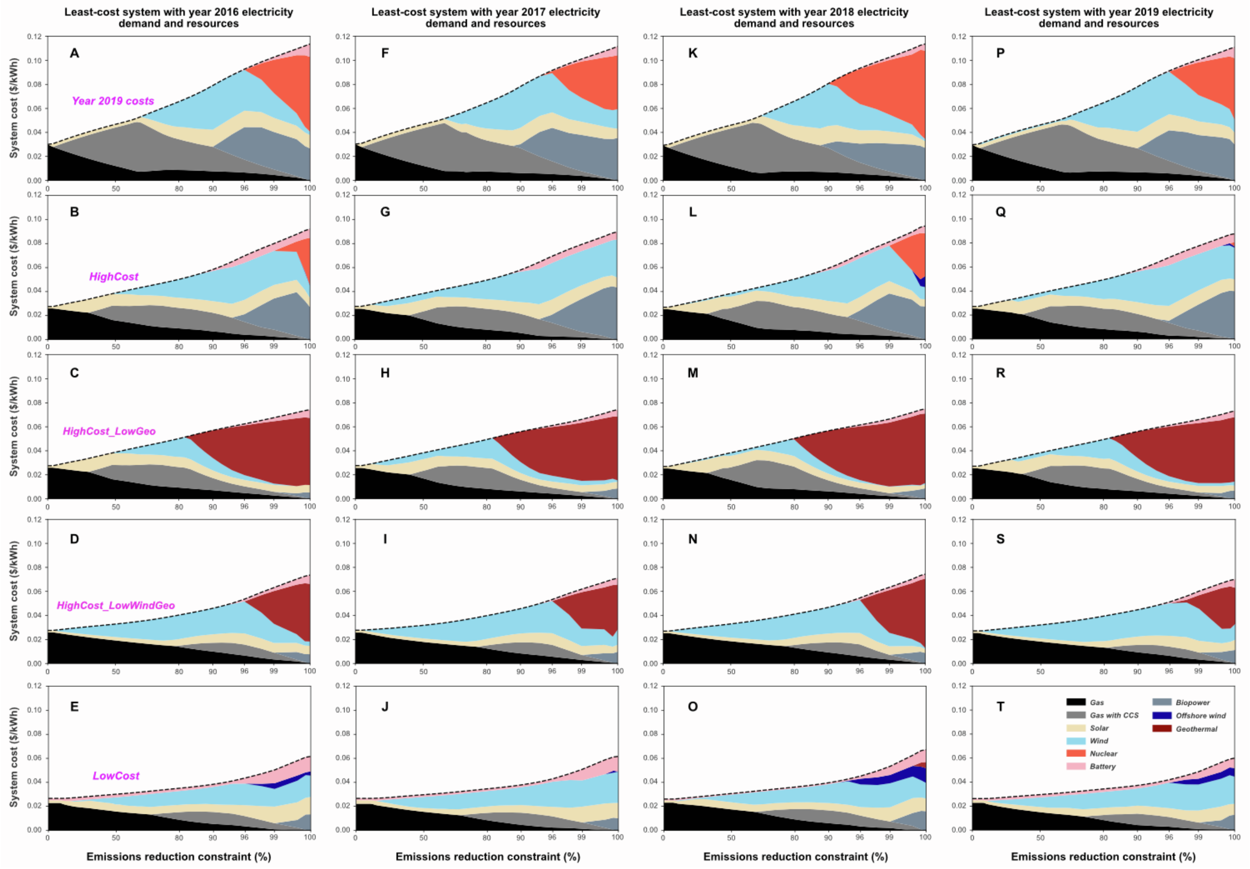

Supplementary Figure S5. Technology costs from the National Renewable Energy Laboratory (NREL) 2021 Annual Technology Baseline (ATB) report, related to STAR Methods. Panel (**A**) shows the % ratio between the projected year-2050 and year-2019 fixed costs, and panel (**B**) shows costs to produce one-kilowatt-hour (kWh) of electricity for different technologies. That is, panel (**B**) shows fixed cost plus variable cost for gas, gas-with-CCS, and biopower, fixed cost for nuclear and geothermal power, and fixed cost divided by annual mean capacity factor for onshore wind, offshore wind, and solar.

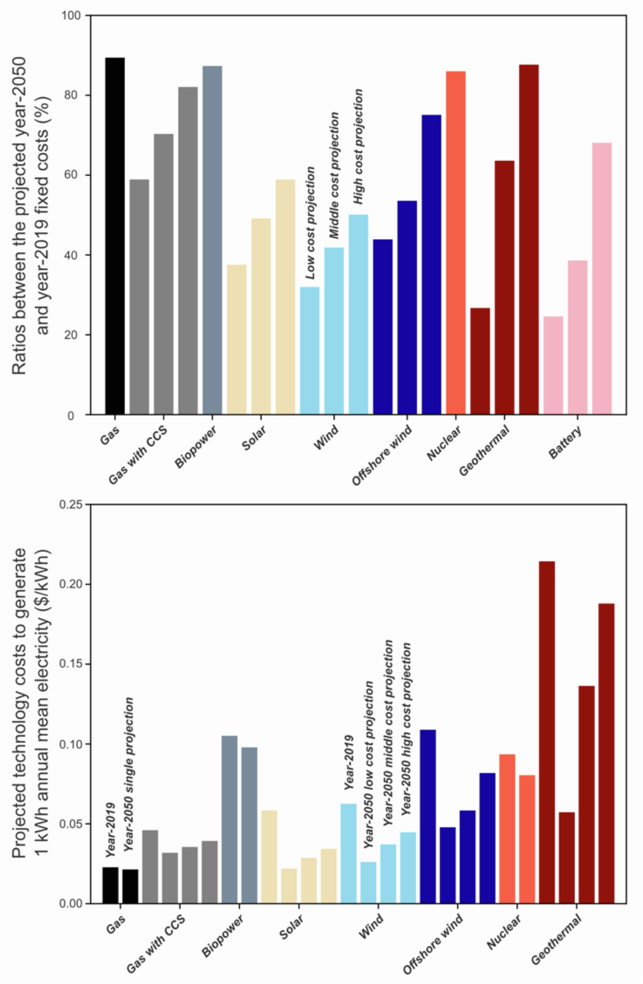

Supplementary Figure S6. Daily and hourly Electricity dispatch, related to Figure 3. (**A-D**) 10%, (**E-H**) 20%, and (**I-L**) 50% emission reduction constraints are compared for four representative year-2050 cost combination cases (i.e., **HighCost**, **HighCost_LowGeo**, **HighCost_LowWindGeo**, and **LowCost**). Rows correspond to the four panels in Figure 2.

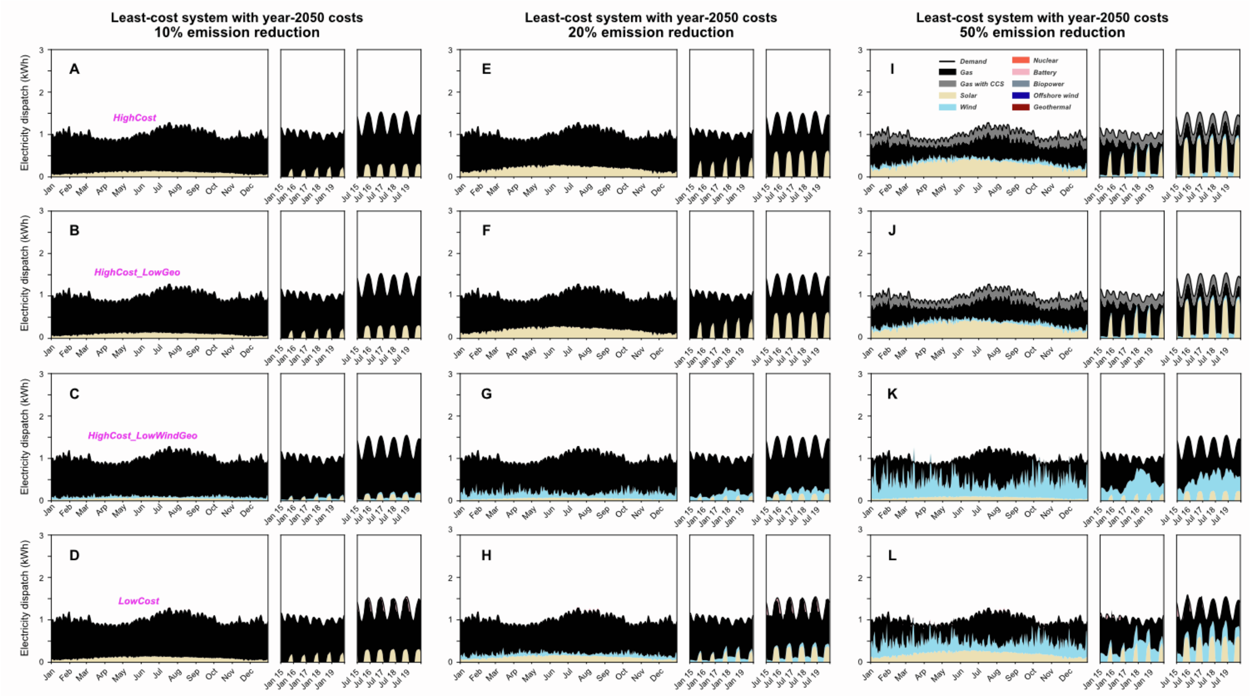

Supplementary Figure S7. Distribution of simulated costs and capacities, related to Figure 4. Similar to Figure 4 but under the 99% emission reduction constraint, in which fossil fuel sources (i.e., gas and gas-with-CCS) exist in technology mixes as well (in total 729 cases).

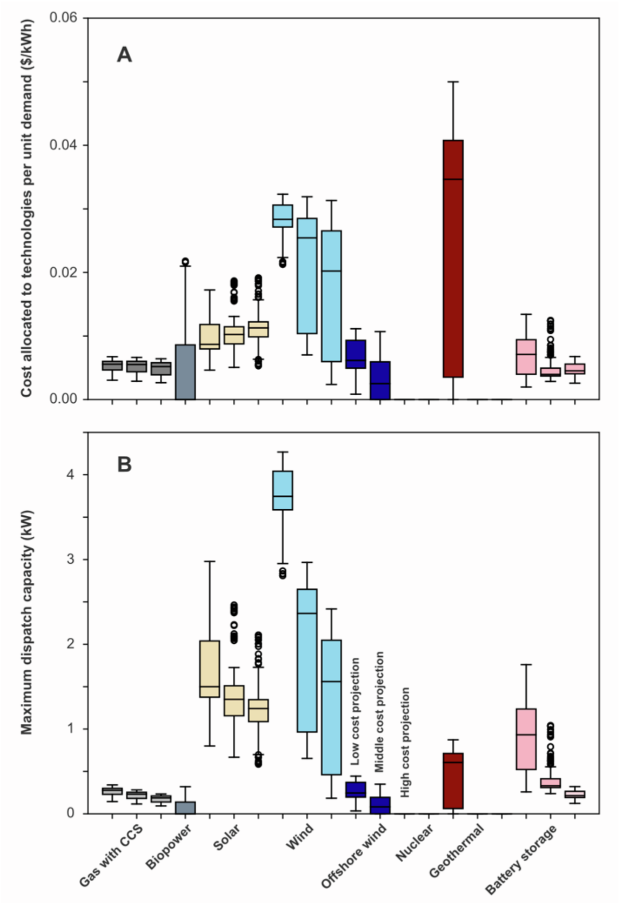

Supplementary Figure S8. Competition between the onshore wind and geothermal, related to Figure 4. Panel (**A**) shows the model simulated geothermal capacity versus onshore wind capacity and panel (**B**) shows generation potential across different ensemble members under the 100% emission reduction constraint. Annual mean demand is normalized so that the mean value is 1 kWh per hour. Cases with zero geothermal capacity are not included.

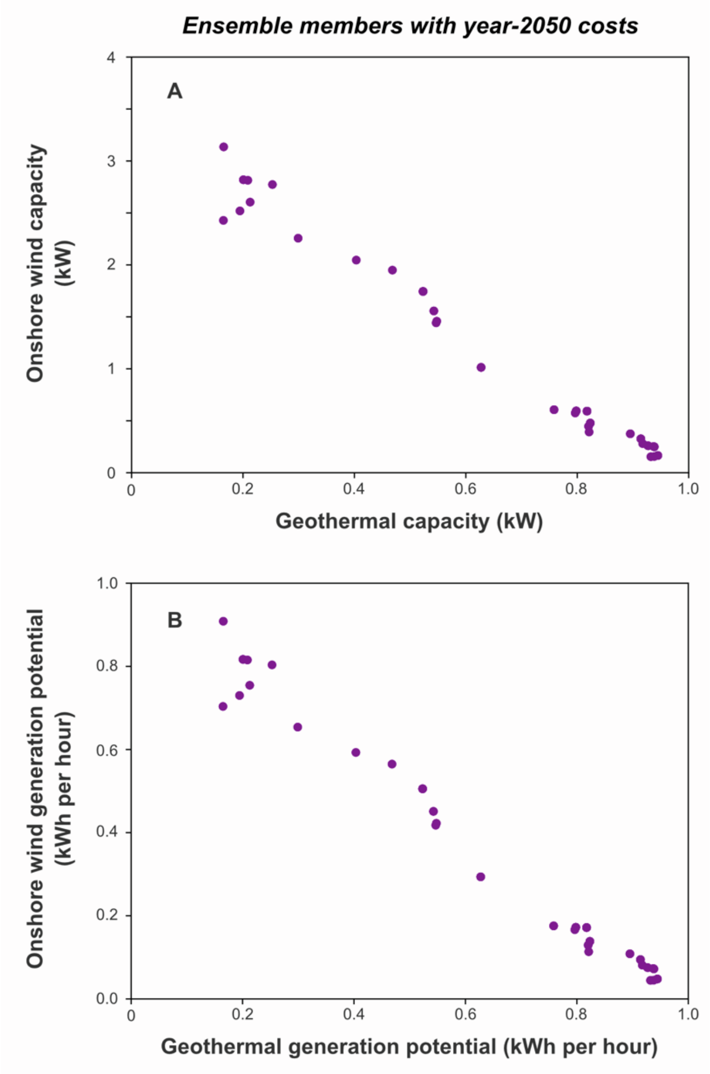

Supplementary Figure S9. Price elasticity of demand, related to Figure 4. Panel (**A**) shows price elasticity of demand calculated as ratios between percentage increases in installed capacity for each percentage decreases in fixed costs in each technology, considering the full range of costs in competing technologies. Panel (**B**) shows the same results, but in absolute value instead of percentage change. Each technology has two bars, showing elasticity from middle to low, and high to middle, future cost projection levels. A value of 10 in panel (**A**) means that a 1% reduction in technology cost would lead to a 10% increase in deployment of that technology.

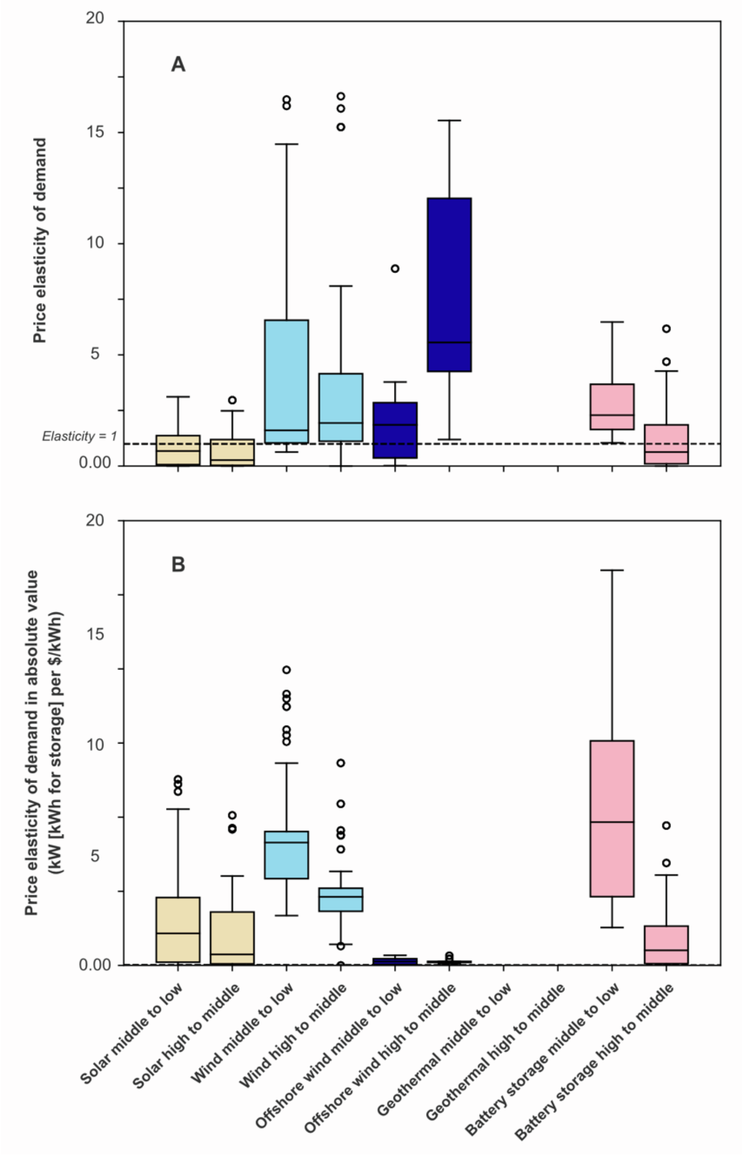

Supplementary Figure S10. Distribution of ensemble members, related to Figure 5. Similar to Figure 5 but under the 99% emission reduction constraint, in which fossil fuel sources (i.e., gas and gas-with-CCS) exist in technology mixes as well (in total 729 cases).

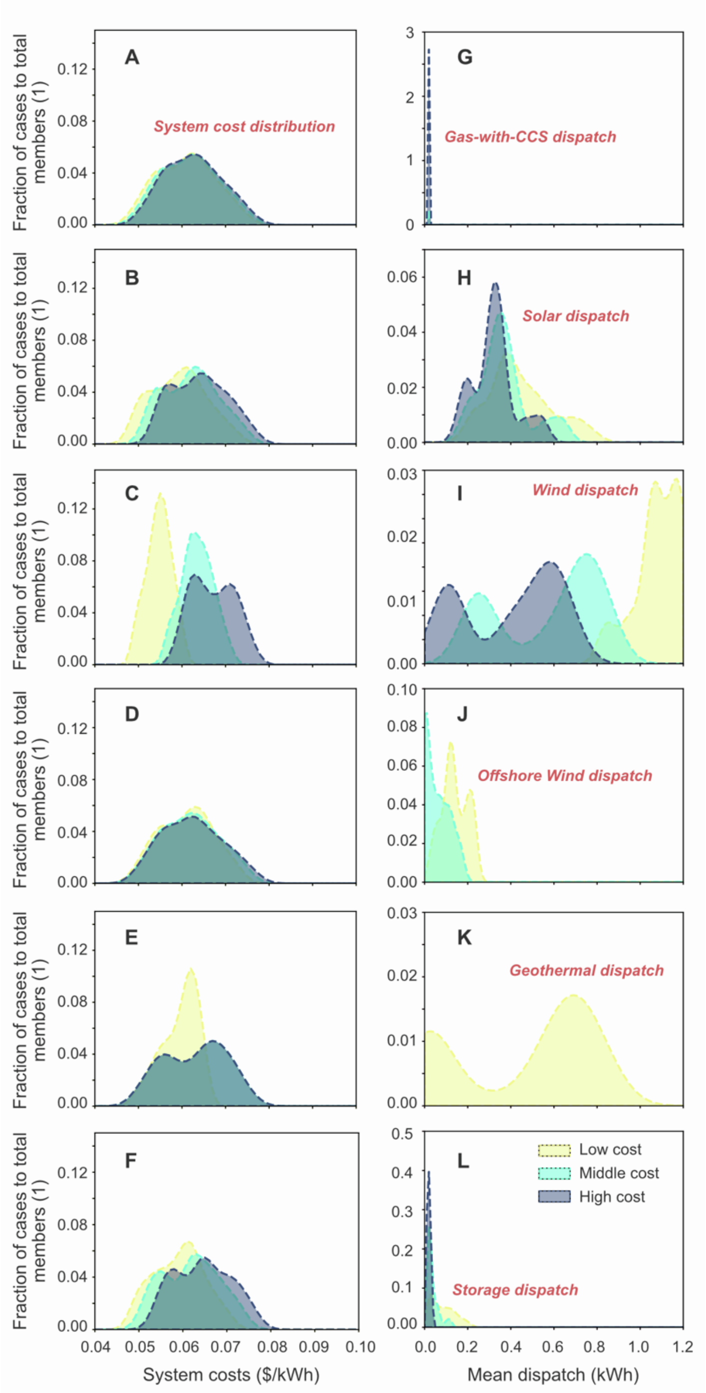

Supplementary Figure S11. Distribution of system cost under different technology cost levels, related to Figure 5. (**A**) For each technology, dots represent the distribution of system costs of the least-cost solutions under that cost level. When dots are within the boundary lines, it means technologies are built in corresponding optimized solutions, and vice versa; (**B**) Mean and one standard deviation of system costs under different technology cost levels. Different colors represent different technologies and markers show various cost levels. Compared to other technologies, cost reductions in wind and geothermal not only decrease overall system costs, but also reduce the uncertainty of system cost distributions.

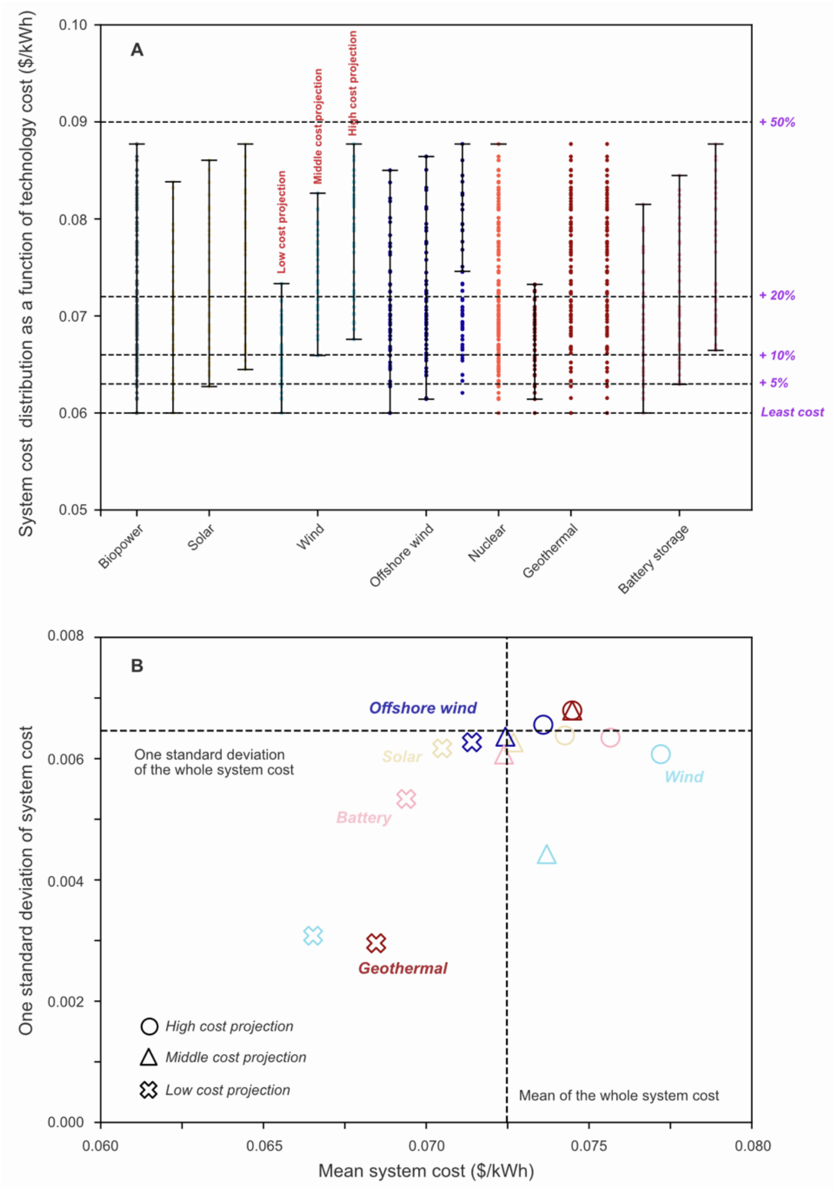

Supplementary Figure S12. Impact of restricting the maximum allowable geothermal capacity on system costs, related to Figure 5. Here we show under the 100% emission reduction constraint, the distribution of system cost, calculated as the ratio of cases to total ensemble members under different system cost levels. The maximum allowable geothermal capacity is set at various levels (e.g., **A** no constraint, **B** 0.7 kW, **C** 0.5 kW, and **D** 0.3 kW, where annual mean demand is normalized to 1 kWh per hour).

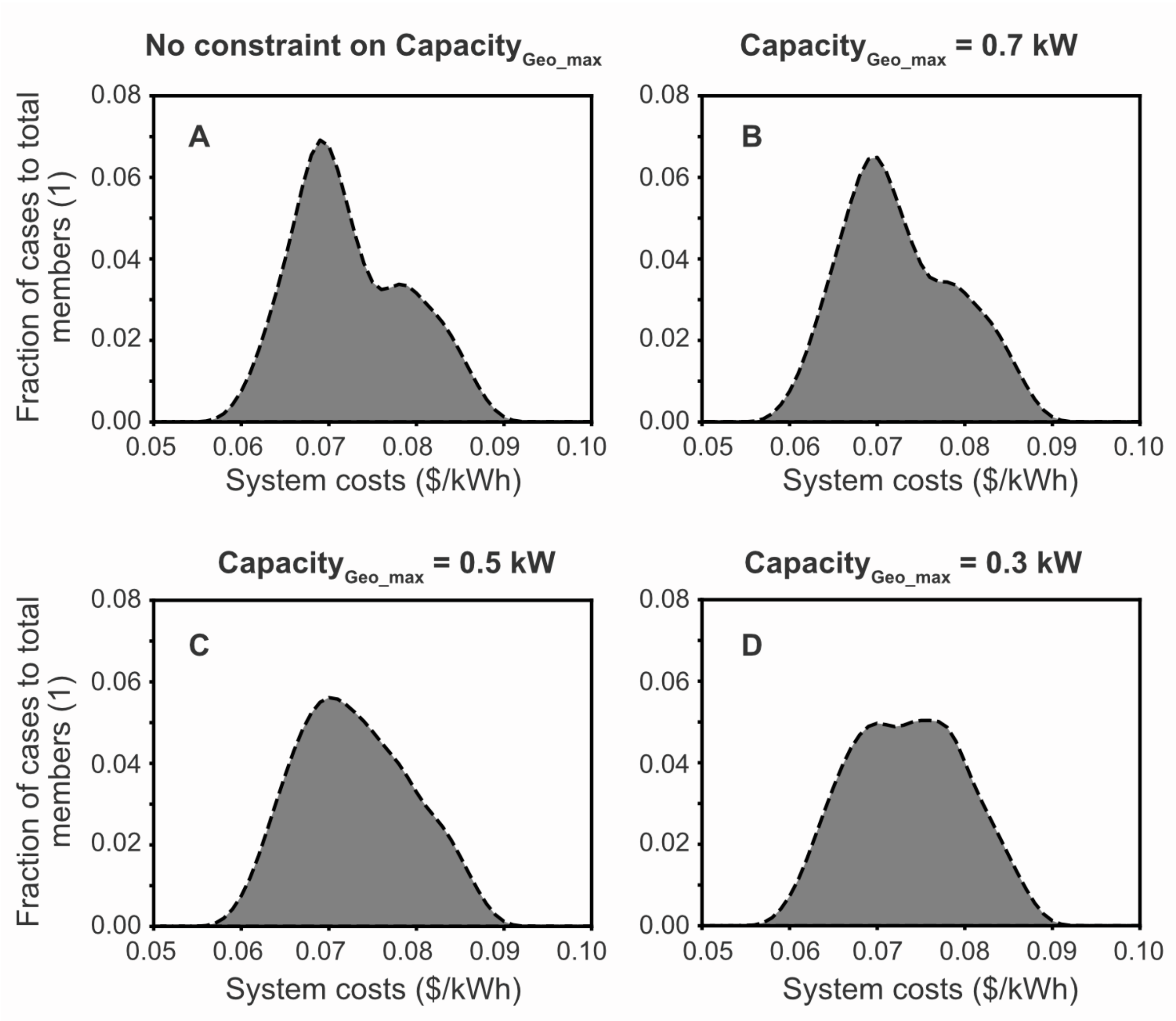

Supplementary Figure S13. Impact of restricting the maximum allowable geothermal capacity on system costs, related to Figure 5. Similar to Figure 5 but here we set the maximum allowable geothermal capacity to various levels (e.g., **A**-**E** no constraint, **F**-**J** 0.7 kW, **K**-**O** 0.5 kW, and **P**-**T** 0.3 kW, where annual mean demand is normalized to 1 kWh per hour).

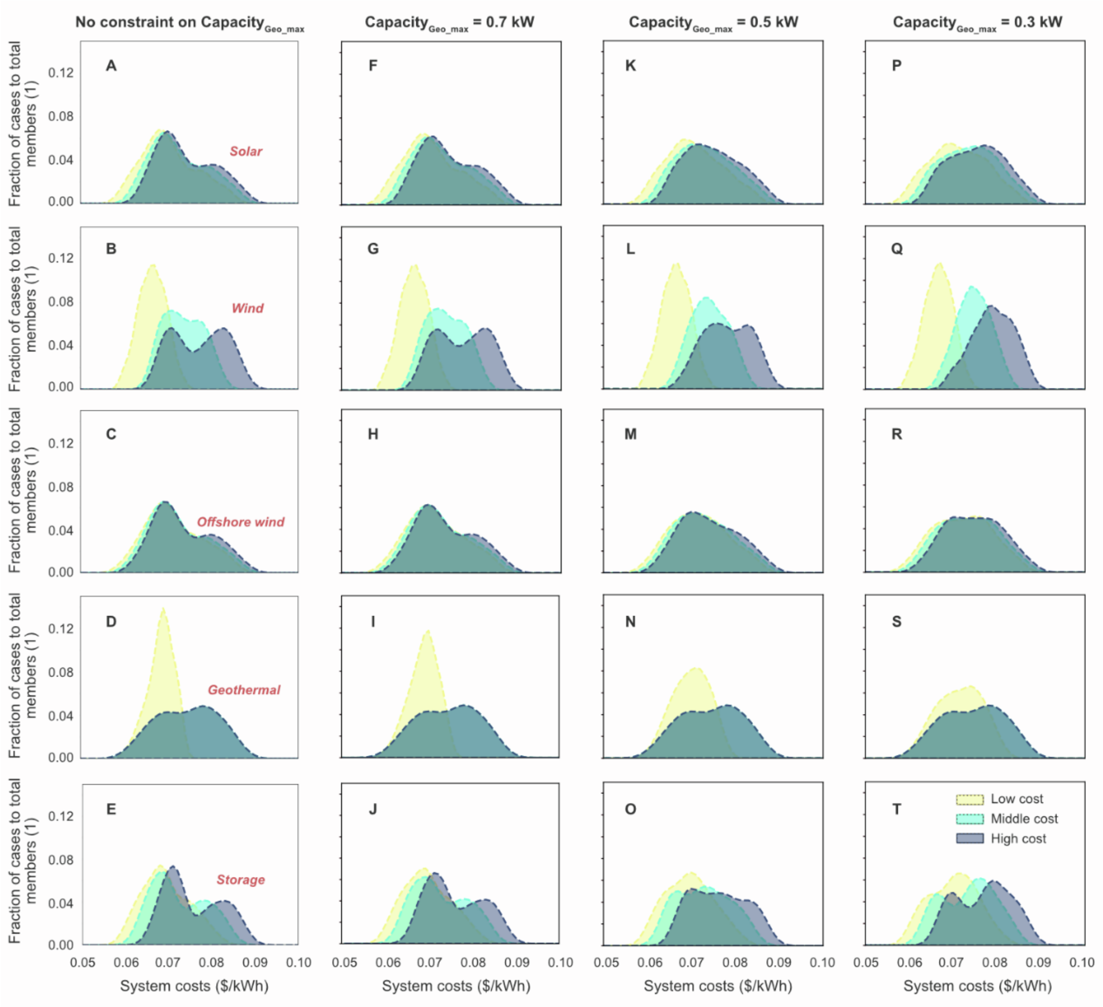

Supplementary Figure S14. Comparisons of system cost distributions, related to STAR Methods. Panel (**A**) shows system cost distributions from the single-year-2050 ensemble simulations, in which the year-2050 technology cost estimates are used and there is no pre-fixed capacity for all technologies; panel (**B**) shows the transient-year-2050 system cost distributions from the transient simulations, in which simulations start from year 2019 to year 2050. Emission reduction constraint increases linearly from 0% to 100% and technology costs are updated each year based on the National Renewable Energy Laboratory (NREL) 2021 Annual Technology Baseline (ATB) report

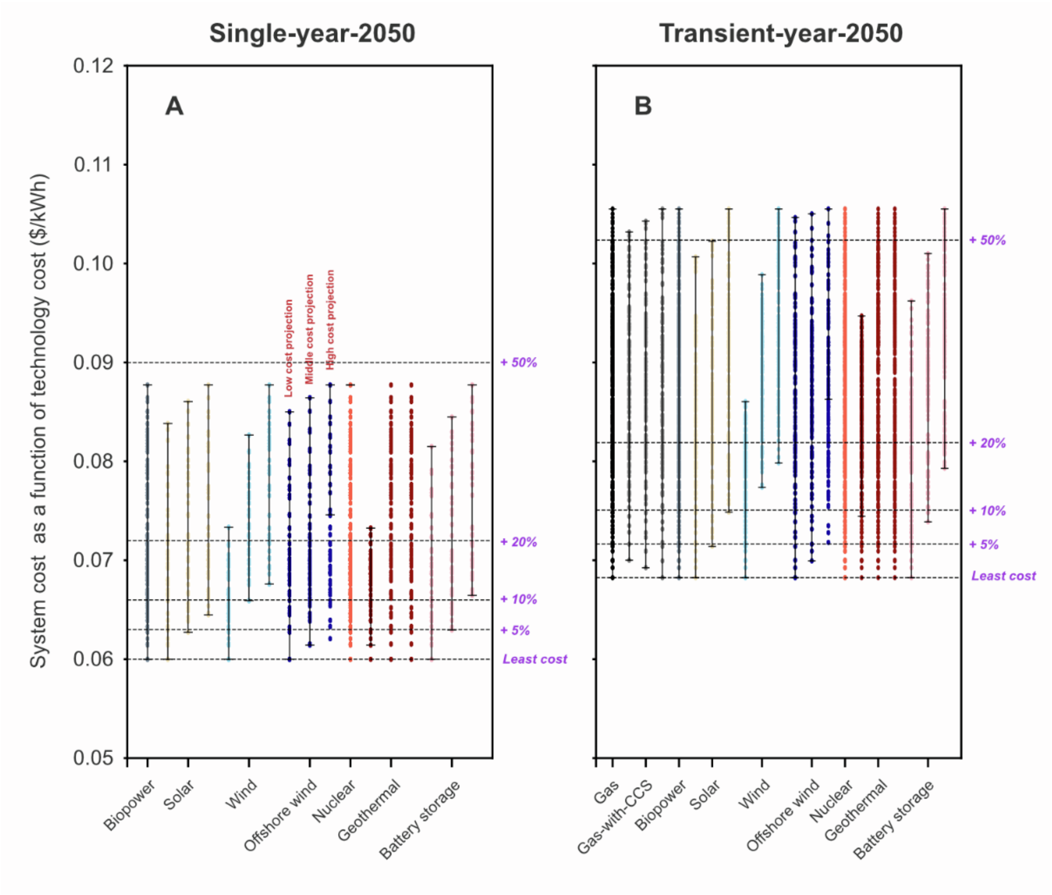

153 **Supplementary Figure S15.** Distribution of the transient-year-2050 ensemble members results,
154 related to STAR Methods.

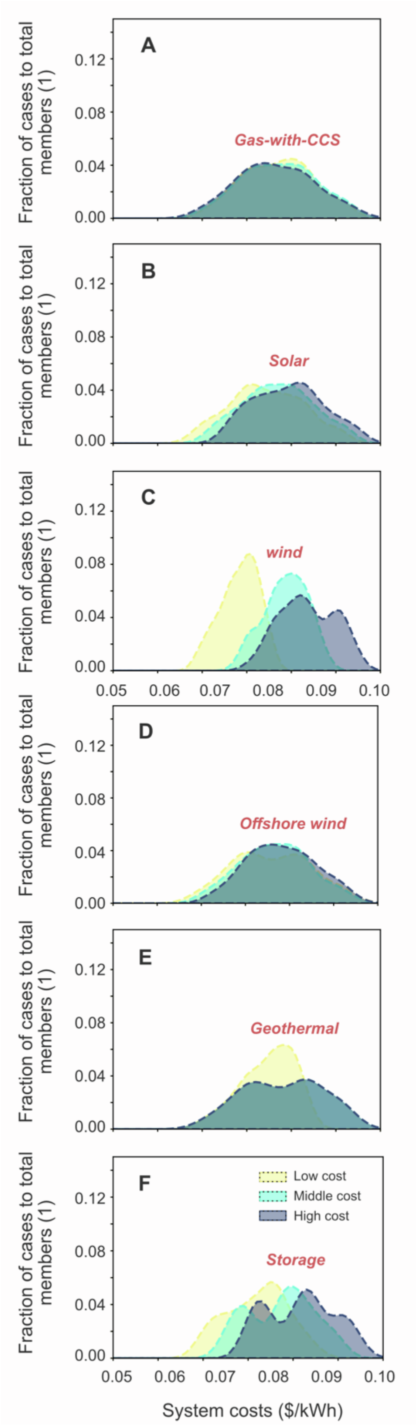

155

Supplementary Figure S16. Difference in simulated technology capacities, related to STAR Methods. Differences between the year-2050 results from transient simulations and single-year greenfield simulations are shown.

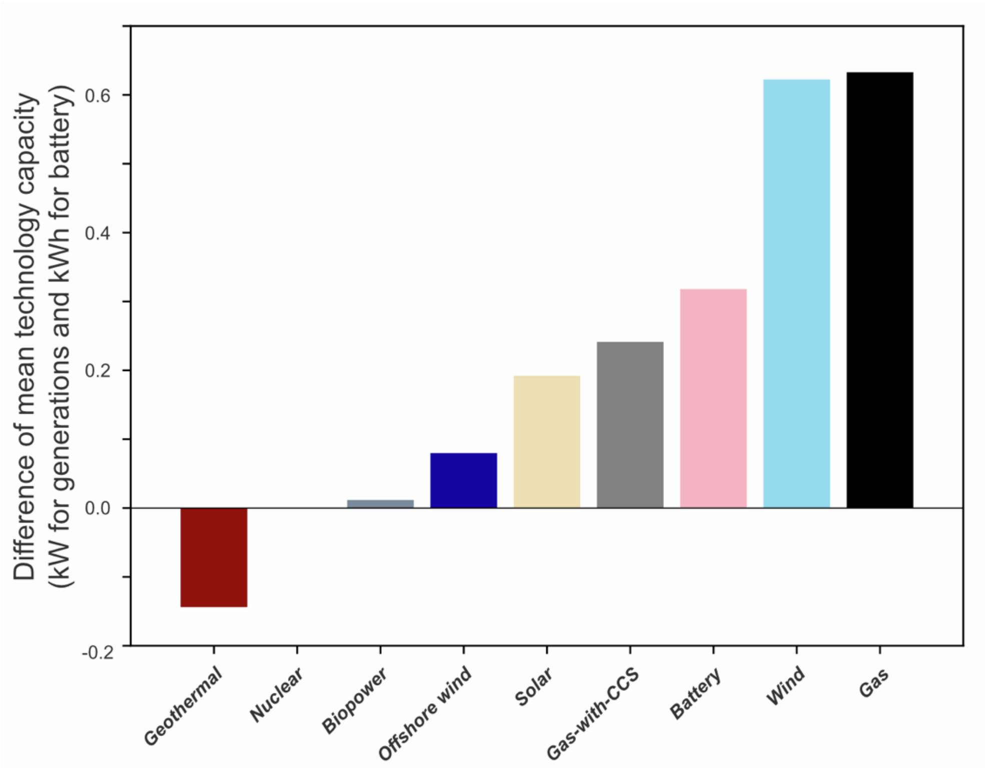

Supplementary Figure S17. Contributions to difference in actual system cost, related to Figure 6. For a given technology capacity mix under a decarbonized future and 243 possible year-2050 cost combinations, here we show contributions from various technology components to difference in actual costs between two cases: one that has the highest actual cost and the other has the lowest actual cost among all 243 possibilities. Results for different given technology capacity mixes are shown ranked by their mean actual costs.

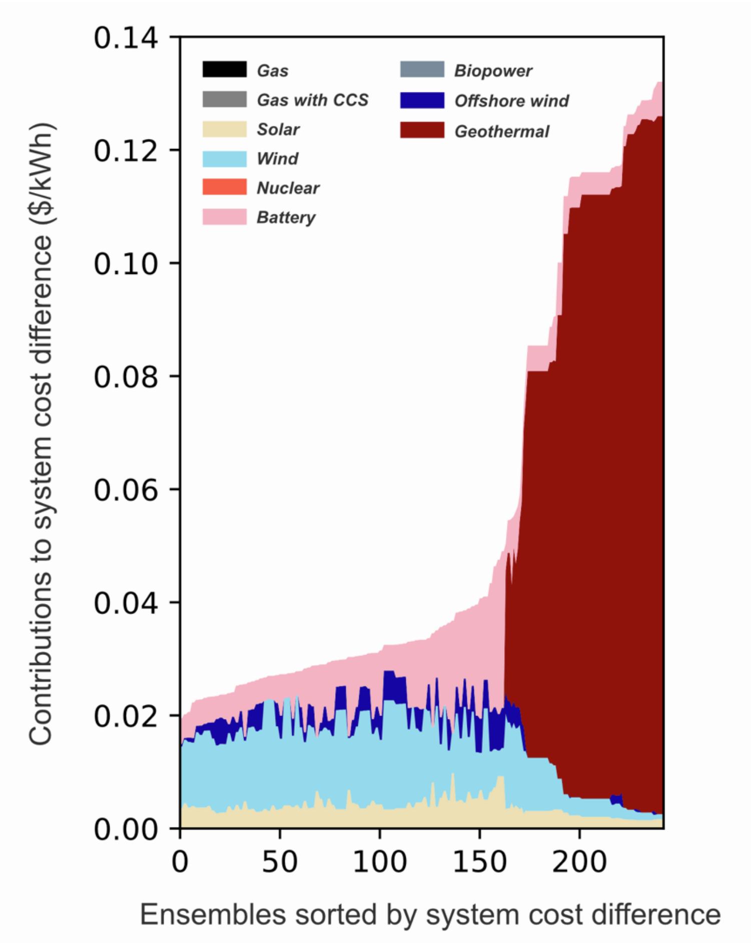

Supplementary Figure 18. Additional cases with lower solar, wind, and battery costs, related to Figure 5. Here we show similar figures to Figure 5, but with cases where costs for solar, wind, and battery are 30%, 50%, and 70% of the **LowCost** levels. Compared to cases using the NREL 2021 ATB cost levels, these cases have more dispatch from these technologies and lower system costs.

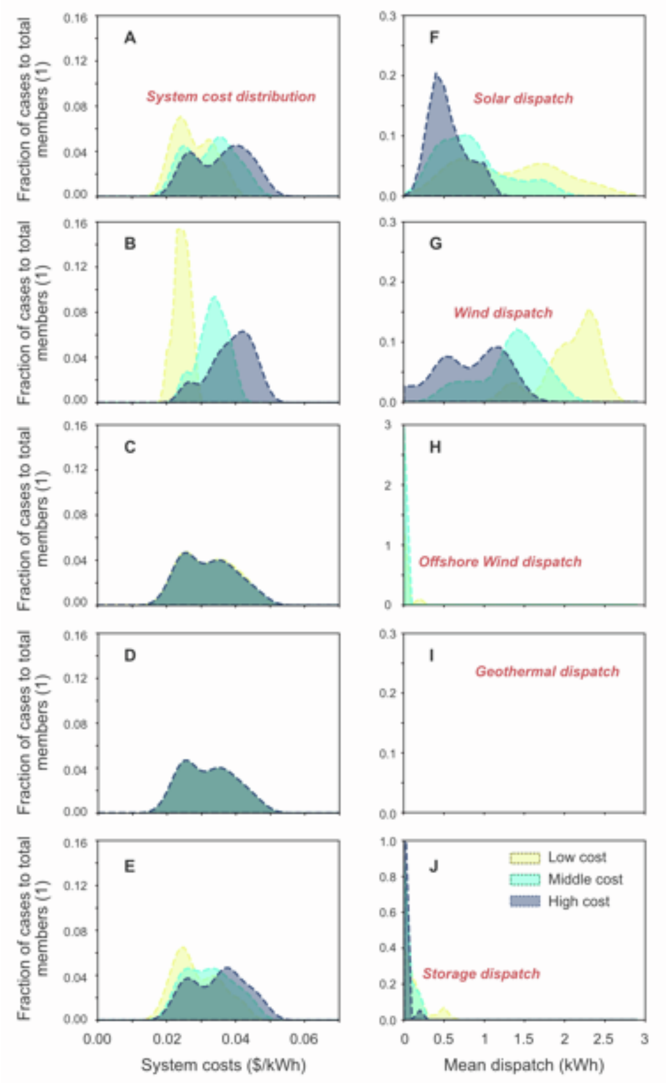

Supplementary Figure 19. Expected system cost and uncertainty in actual cost, related to Figure 6. Similar to Figure 6 but with cases where costs for solar, wind, and battery are 30%, 50%, and 70% of the **LowCost** levels.

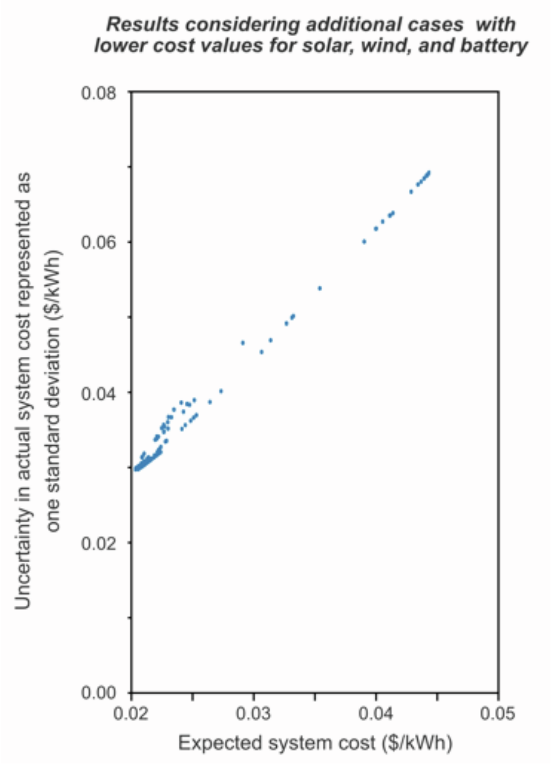

Supplementary Figure 20. Uncertainty of long-duration storage, related to STAR Methods. Long-duration storage here is represented by a hydrogen storage system, which has three separate parts in the model (the electrolyzer, which produces H_2 using electricity; H_2 storage, which stores the produced H_2 underground, and the full cell, which converts H_2 back to electricity). Here we scale the electrolyzer and full cell together (panel **A-D**) or separately (panel **E-L**) to examine their impacts on systems under the 100% emission reduction constraints. For a scale of 1, the fixed cost is \$0.023/kWh for the electrolyzer, representing near-current cost estimates for electrolyzer. The fixed costs for the full cell are \$0.058/kWh for a scale of 1 and \$0.023/kWh for a scale of 0.4, representing near-current cost estimates for different types of fuel cells.

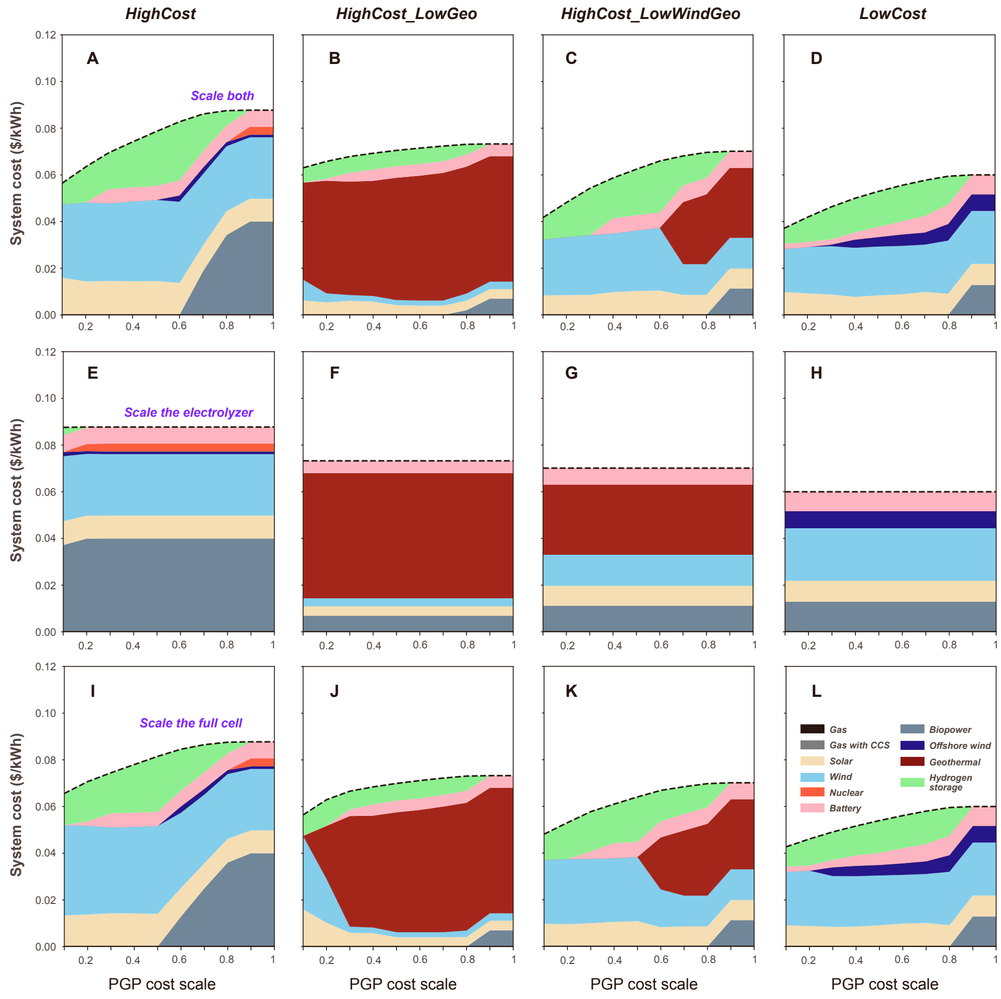

Supplementary Figure S21. Uncertainty of direct air capture (DAC), related to STAR Methods. DAC is represented to be able to remove atmospheric CO_2 at a cost varying from \$10/tCO_2 to \$1000/tCO_2. Different year-2050 cost combination cases (**A-D**) are compared.

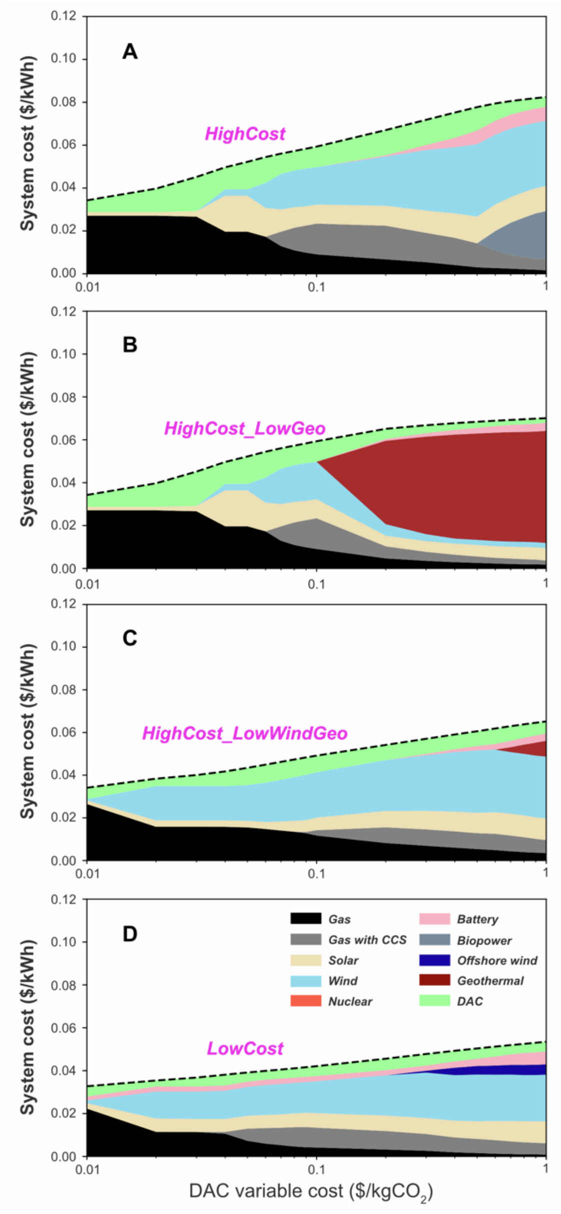

Supplementary Figure S22. New cost estimates, related to STAR Methods. Comparisons of the fixed-cost estimates based on the National Renewable Energy Laboratory (NREL) 2021 Annual Technology Baseline (ATB) report and that from NREL 2022 ATB report version 3.

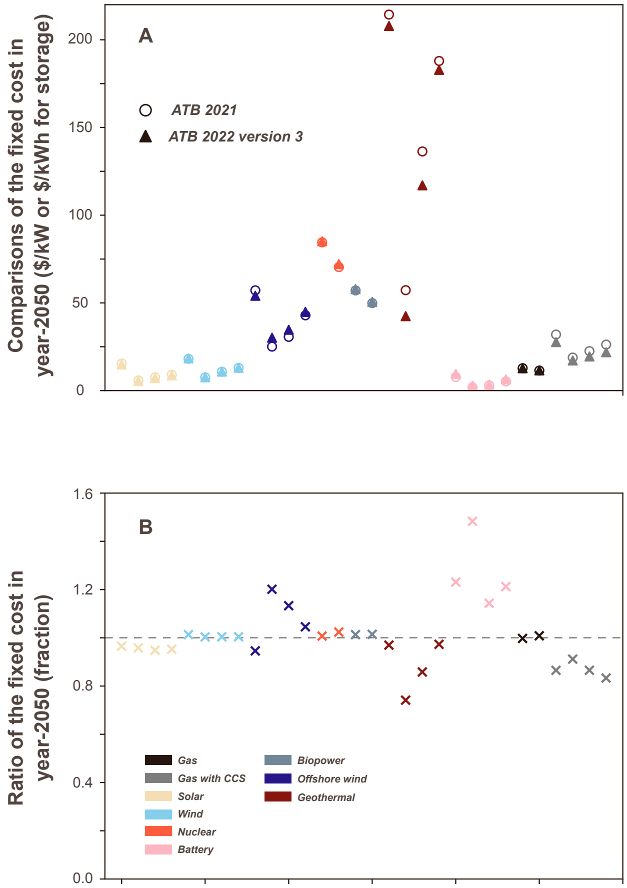

Supplementary Figure S23. Impact of a more variant electricity demand profile, related to STAR Methods. Panels **A-E** use hourly electricity demand profile from the NREL Electrification Future Study (EFS) report, and panels **F-J** consider an idealized demand profile calculated as the square of the year-2019 demand from the U.S. Energy Information Administration (EIA). The same year-2019 renewable potentials are combined with different demand profiles for simulations.

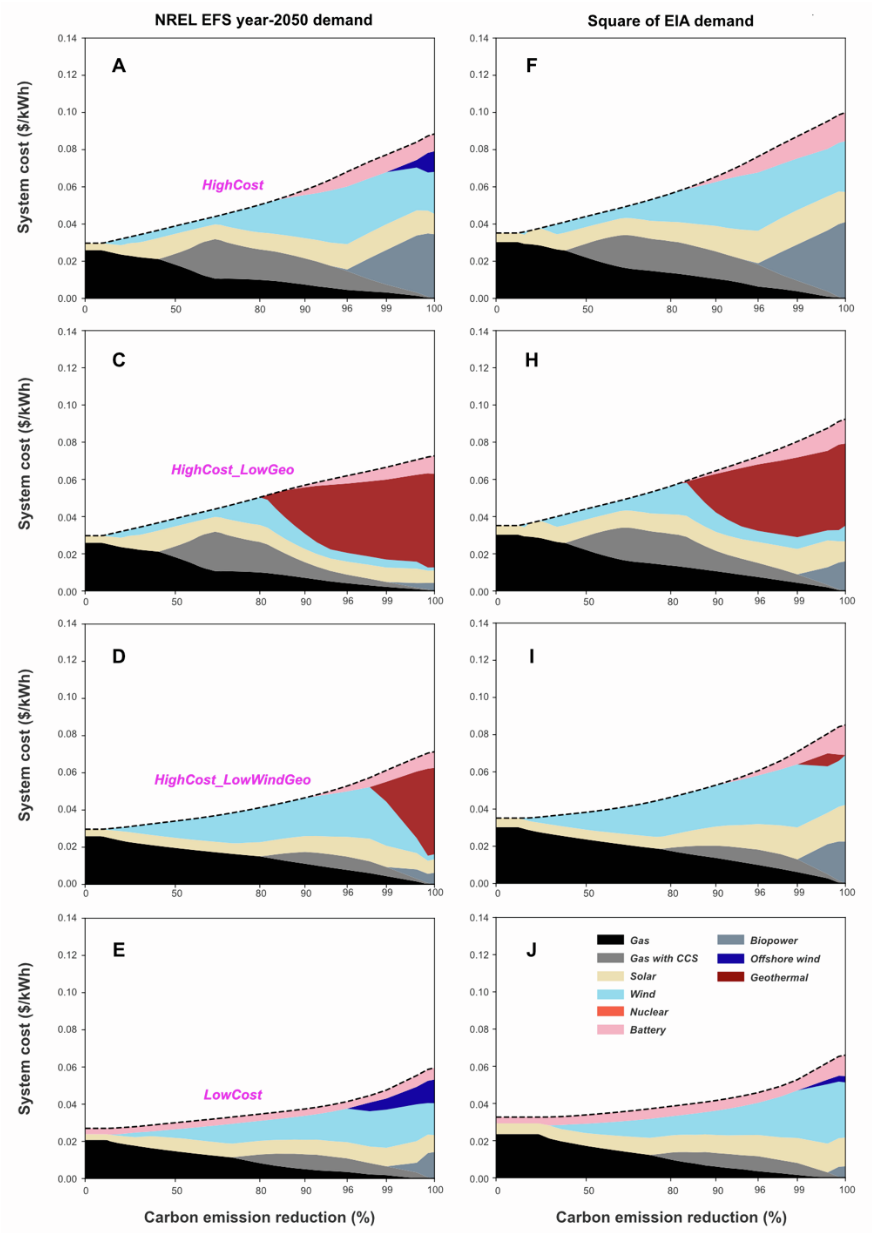

Supplementary Figure S24. Impact of different gas-with-CCS assumptions, related to STAR Methods. Panel (**A**) shows the default simulation results under the year-2019 cost levels, similar to Figure S1, panel (**B**) shows cases with more expensive variable costs for gas and gas-with-CCS, panel (**C**) shows cases with a lower CO_2 emission rate per kWh electricity generated (i.e., higher capture rate for CO_2, ~ 90%) for gas-with-CCS, and panel (**D**) shows the combined effect of more expensive variable costs and higher CO_2 capture rate.

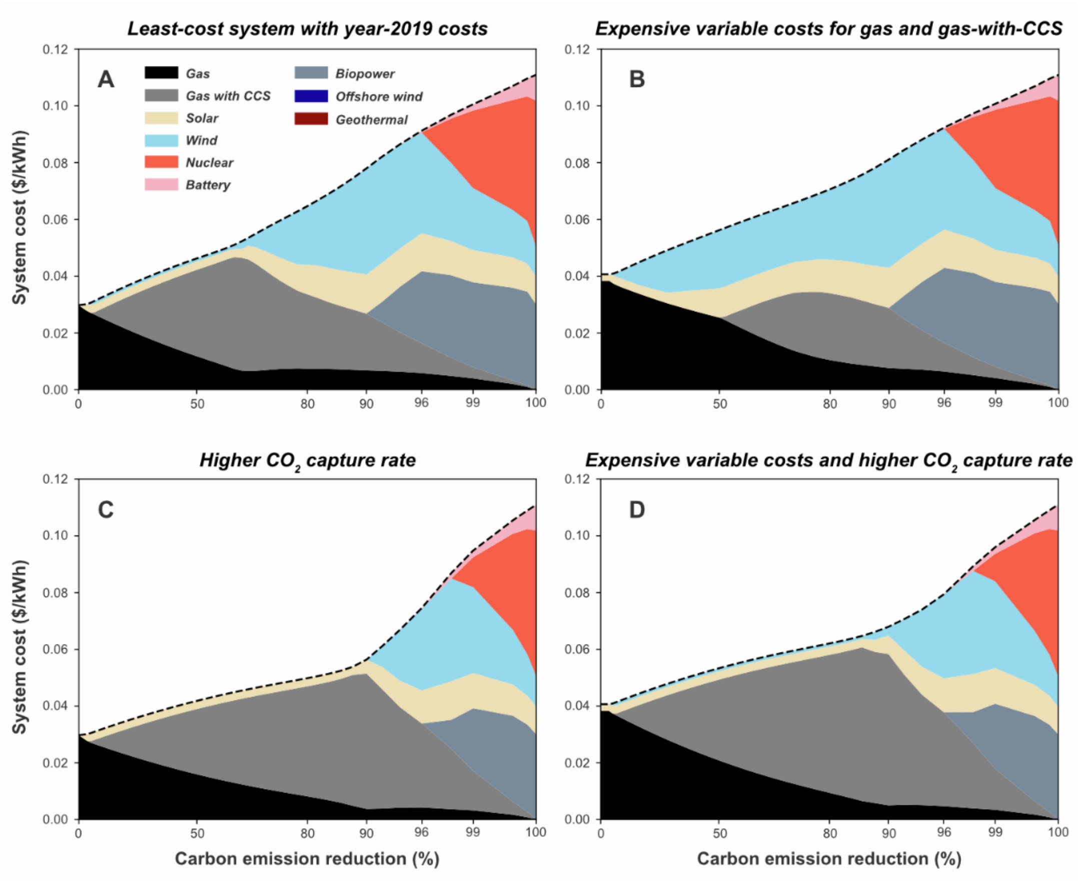

Supplementary Table S1. Cost assumptions, related to Figure 1. Costs are taken from the National Renewable Energy Laboratory (NREL) 2021 Annual Technology Baseline (ATB) report. All dollar values are represented in year-2019 dollar. Capacity lifetime is assumed to be 15 years for battery and 30 years for others. The default discount rate used is 0.07.

		**Capital expenditures ($/kW, $/kWh for battery)**	**Fixed O&M cost ($/kW-year)**	**Hourly fixed cost ($/MW)[1]**	**Variable O&M cost ($/MWh)**	**Fuel cost ($/MWh)**	**Variable cost ($/MWh)**
**Gas[2]**	Year-2019	1054	27	12.78	2	8	10
	Year-2050	907	27	11.43	2	8	10
**Gas-with-CCS[3]**	Year-2019	2670	65	31.98	6	8	14
	Year-2050	1353/1700/2047	56/60/65	18.84/22.49/26.25	5/5/6	8	13/13/14
**Biopower**	Year-2019	4346	150	57.10	5	43	48
	Year-2050	3560	150	49.87	5	43	48
**Solar**	Year-2019	1391	23	15.42	N/A	N/A	N/A
	Year-2050	481/638/776	12/15/17	5.79/7.58/9.08	N/A	N/A	N/A
**Wind**	Year-2019	1436	43	18.13	N/A	N/A	N/A
	Year-2050	525/760/900	24/33/41	7.57/10.76/12.96	N/A	N/A	N/A
**Offshore wind**	Year-2019	5183	83	57.16	N/A	N/A	N/A
	Year-2050	2181/2695/3821	44/51/68	25.09/30.61/42.91	N/A	N/A	N/A
**Nuclear**	Year-2019	7388	145	84.52	2	7	9
	Year-2050	5856	145	70.42	2	8	10
**Geothermal**	Year-2019	19977	268	214.37	N/A	N/A	N/A
	Year-2050	4411/12042/17102	146/224/268	57.25/136.35/187.92	N/A	N/A	N/A
**Battery**	Year-2019	290	36	7.74	N/A	N/A	N/A
	Year-2050	70/102/193	9/15/25	1.90/2.99/5.27	N/A	N/A	N/A

[1]Hourly fixed cost (C_{fixed_cost}) is calculated as

$$C_{fixed\_cost} = C_{capital\_cost} * CRF + C_{O\&M}$$

where $C_{capital\_cost}$ represents the overnight capital cost, $C_{O\&M}$ represents the fixed O&M cost, and CRF represents the capital recovery factor, which is calculated based on the discount rate R and lifetime N:

$$CRF = \frac{R * (1+R)^N}{(1+R)^N - 1}$$

[2]Lifecycle emissions considered for gas is 0.49 $kgCO_2$ per kWh electricity generated.

[3]Lifecycle emissions considered for gas-with-CCS is 0.17 $kgCO_2$ per kWh electricity generated.

Supplementary Table S2. Contributions of different technologies to total system costs, related to Figure 4. Values are calculated as costs associated with different technology components divided by the total system cost in percentage units. The minimum, maximum, and one standard deviation values are shown. The 100% emission reduction constraint cases are examined here.

Technology and cost level	Minimum	Maximum	1-standard deviation
Gas	0.00	0.00	0.00
Gas-with-CCS all cost levels	0.00	0.00	0.00
Gas-with-CCS low-cost projection	0.00	0.00	0.00
Gas-with-CCS middle-cost projection	0.00	0.00	0.00
Gas-with-CCS high-cost projection	0.00	0.00	0.00
Biopower	7.42	49.67	12.13
Solar all cost levels	3.87	23.26	4.39
Solar low-cost projection	3.87	23.26	5.11
Solar middle-cost projection	4.72	20.46	4.26
Solar high-cost projection	5.61	19.03	3.60
Wind all cost levels	2.88	41.12	11.79
Wind low-cost projection	17.27	41.12	7.42
Wind middle-cost projection	5.55	38.51	12.11
Wind high-cost projection	2.88	37.08	12.50
Offshore wind all cost levels	0.00	15.33	4.72
Offshore wind low-cost projection	0.00	15.28	3.97
Offshore wind middle-cost projection	0.00	15.33	4.86
Offshore wind high-cost projection	0.00	8.76	2.16
Nuclear	0.00	3.86	0.35
Geothermal all cost levels	0.00	78.79	29.25
Geothermal low-cost projection	0.00	78.79	18.93
Geothermal middle-cost projection	0.00	0.00	0.00
Geothermal high-cost projection	0.00	0.00	0.00
Storage all cost levels	4.32	22.34	3.44
Storage low-cost projection	4.40	22.34	4.67
Storage middle-cost projection	4.32	18.27	3.04
Storage high-cost projection	7.15	11.08	1.11

Supplementary Table S3. Contributions of different technologies to total system costs, related to Figure 4. Same as Table S2 but for 99% emission reduction constraint cases.

Technology and cost level	Minimum	Maximum	1-standard deviation
Gas	1.06	5.43	0.83
Gas-with-CCS all cost levels	4.24	12.54	2.27
Gas-with-CCS low-cost projection	4.75	12.54	2.25
Gas-with-CCS middle-cost projection	4.66	12.03	2.23
Gas-with-CCS high-cost projection	4.24	11.53	2.23
Biopower	0.00	28.74	8.14
Solar all cost levels	7.28	28.33	5.47
Solar low-cost projection	7.28	27.83	5.70
Solar middle-cost projection	7.80	28.33	5.46
Solar high-cost projection	8.16	27.22	5.22
Wind all cost levels	3.63	60.64	15.40
Wind low-cost projection	42.34	60.64	4.39
Wind middle-cost projection	10.83	48.57	12.73
Wind high-cost projection	3.63	43.60	13.31
Offshore wind all cost levels	0.00	16.56	5.64
Offshore wind low-cost projection	1.28	16.56	3.98
Offshore wind middle-cost projection	0.00	14.22	4.57
Offshore wind high-cost projection	0.00	0.00	0.00
Nuclear	0.00	0.00	0.00
Geothermal all cost levels	0.00	76.08	25.86
Geothermal low-cost projection	0.00	76.08	29.00
Geothermal middle-cost projection	0.00	0.00	0.00
Geothermal high-cost projection	0.00	0.00	0.00
Storage all cost levels	3.14	22.11	4.30
Storage low-cost projection	3.14	22.11	5.28
Storage middle-cost projection	4.45	18.71	3.41
Storage high-cost projection	4.03	12.25	1.93

Reference

1. Duan, L., Petroski, R., Wood, L. & Caldeira, K. Stylized least-cost analysis of flexible nuclear power in deeply decarbonized electricity systems considering wind and solar resources worldwide. *Nat. Energy* (2022) doi:10.1038/s41560-022-00979-x.
